# A single mini-barcode test to screen for Australian mammalian predators from environmental samples

**DOI:** 10.1093/gigascience/gix052

**Published:** 2017-07-04

**Authors:** Elodie Modave, Anna J MacDonald, Stephen D Sarre

**Affiliations:** Institute for Applied Ecology, University of Canberra, ACT, 2601, Canberra, Australia

**Keywords:** 12S rRNA, dasyurus, DNA barcoding, DNA detection, marsupial, monitoring

## Abstract

Identification of species from trace samples is now possible through the comparison of diagnostic DNA fragments against reference DNA sequence databases. DNA detection of animals from non-invasive samples, such as predator faeces (scats) that contain traces of DNA from their species of origin, has proved to be a valuable tool for the management of elusive wildlife. However, application of this approach can be limited by the availability of appropriate genetic markers. Scat DNA is often degraded, meaning that longer DNA sequences, including standard DNA barcoding markers, are difficult to recover. Instead, targeted short diagnostic markers are required to serve as diagnostic mini-barcodes. The mitochondrial genome is a useful source of such trace DNA markers because it provides good resolution at the species level and occurs in high copy numbers per cell. We developed a mini-barcode based on a short (178 bp) fragment of the conserved 12S ribosomal ribonucleic acid mitochondrial gene sequence, with the goal of discriminating amongst the scats of large mammalian predators of Australia. We tested the sensitivity and specificity of our primers and can accurately detect and discriminate amongst quolls, cats, dogs, foxes, and devils from trace DNA samples. Our approach provides a cost-effective, time-efficient, and non-invasive tool that enables identification of all 8 medium-large mammal predators in Australia, including native and introduced species, using a single test. With modification, this approach is likely to be of broad applicability elsewhere.

## Background

The looming biodiversity crisis, referred to by some as the “Sixth Mass Extinction” [1], has made the conservation of wildlife a rapidly growing concern. There is an urgent need to document the distribution of biodiversity as the foundation for identifying effective solutions to wildlife management issues. The rapid and reliable identification of species at local and regional scales can provide the first step toward determining the distribution of biodiversity in the landscape and changes that might be occurring in that distribution.

Advances in genetics and genomics have revolutionized many areas of biology, and in particular, the identification of wildlife from trace and environmental samples (e.g., water, soil, and faeces, or scats) is now possible through DNA barcoding [2–5], where the identity of an unknown sample is established by comparing DNA sequences obtained from that sample to an appropriate reference sequence database. The application of DNA barcoding for the identification of species from such environmental DNA (eDNA) samples is useful, particularly when the target species is rare, elusive, difficult to trap or observe without direct interference with live animals, or where morphological identification is problematic [6–10]. It also makes possible the identification of diet from scats where morphological determinations are likely to be unsuitable for many elements of the diet [11–15]. Consequently, eDNA analysis from environmental samples collected across a broad spatial and temporal distribution has great potential for enhancing biodiversity management but is yet to be widely implemented [16, 17].

The DNA associated with environmental samples tends to be of low quantity or quality and can be degraded. To ensure that markers for eDNA detection are specific and sensitive, target sequences, also known as mini-barcodes, should be short (i.e., 100–200 base pairs [bp]) [2, 18–20] and yet have high discriminatory power [21–24]. Marker selection therefore needs to account for the range of species likely to be encountered, as well as discriminating among potential sister taxa. Mitochondrial DNA genes (mtDNA) are usually targeted because they occur in multiple copies in each cell and are therefore more common in trace samples than nuclear sequences because they can give good resolution of identification at the species level and because their genome is circular, which helps to preserve the DNA in some instances. In regions where little is known of the genetic characteristics of the faunal assemblage, identifying the most appropriate DNA sequences to target the fauna present to achieve acceptable levels of accuracy is a challenging exercise and requires a reference database that is sufficiently comprehensive to ensure accurate species assignment [25]. In short, we need DNA barcoding markers that are appropriate to the question being addressed, the ecosystem considered, and the taxonomic group studied. Most importantly, if DNA detection is going to be of practical benefit, we need to maximize its effectiveness by developing mini-barcodes that target as many taxa as possible, thus minimizing the number of tests that need to be applied. Most DNA barcode studies so far that were implemented for detection of specific species from terrestrial systems have targeted single species (examples in [7, 9, 26, 27]) to avoid the ambiguity that might arise by attempting to simultaneously identify multiple closely related taxa. Here, we tackle this problem using all extant medium-large Australian mammalian predators as a case study.

Australia has a unique assemblage of medium-large mammalian predators, including a suite of marsupials of Gondwanan heritage intermixed with relatively recently arrived eutherian mammals introduced by humans [28, 29]. Here, we develop a DNA mini-barcode to discriminate amongst these key predators, with the goal of species identification using eDNA extracted from scats. We targeted the top native marsupial predators that are likely to produce large easily visible scats, including 6 species of quoll (4 Australian and 2 New-Guinean; *Dasyurus maculatus*, *D. viverrinus*, *D. geoffroii*, *D. hallucatus*, *D. albopunctatus*, and *D. spartacus*), the Tasmanian devil (*Sarcophilus harrisii*), and the extinct thylacine (*Thylacinus cynocephalus*), as well as key eutherian mammal predators: the native dingo (*Canis lupus dingo*) and the introduced domestic dog (*Canis lupus familiaris*), red fox, (*Vulpes vulpes),* and domestic cat (*Felis catus*), which are now feral in much of the country. Most of the native marsupial predators have been in decline since, or even before, European settlement in 1788 [30]. Tasmanian devils and the thylacine became extinct on the Australian mainland within the last 3000 to 4000 years [31] but still existed on the island of Tasmania at the time of European settlement. The thylacine has subsequently been hunted to extinction [32, 33] while devil populations have decreased dramatically since the 1990s following the emergence of Devil Facial Tumour Disease [34–36]. Several species of quoll, together with the dingo, have declined in distribution and abundance on the Australian mainland since European settlement from multiple causes, which probably include habitat destruction, hunting, predation by cats and foxes, the spread of cane toads [37–39], and, in the case of dingos, hybridization with domestic dogs. Although declining or extinct on the mainland, substantial populations of the Tasmanian devil, the spotted-tailed quoll (*D. maculatus*), and the eastern quoll (*D. viverrinus*) remain on the island of Tasmania, where they have important ecological roles [40]. However, recent evidence of foxes in Tasmania [41] and potential competition with feral cats [42, 43] compound the issue and have stimulated an urgent need to understand threats to native predator populations and enable effective management.

Two factors generally limit the application of a DNA barcoding approach. First, short diagnostic sequences that encompass the range of species to be targeted are difficult to find and are likely to be specific to a particular faunal assemblage. Second, the full suite of potential target organisms tends to be poorly known in most natural systems, and reference DNA sequences are not available for many wildlife species, necessitating the development of reference libraries to guide marker selection and interpretation of results. Our goal was to develop a mini-barcode that can identify all medium to large mammal predators in Australia in a single analysis, including quolls, to the species level. This has been difficult to achieve using existing genetic markers because of the high levels of sequence conservation observed between quoll species. We compiled a reference tissue collection and identified a mini-barcode based on the conserved 12S ribosomal ribonucleic acid (rRNA) mitochondrial region that discriminated among taxa with minimal variation within species [44, 45]. We evaluate the specificity and sensitivity of this mini-barcode using the framework outlined in Macdonald and Sarre [25] and Macdonald, Sarre, and Dickman [46]. By targeting all extant media to large carnivores in Australia, we aim to produce a mini-barcode that can be applied broadly within continental Australia as well as Tasmania. We demonstrate that despite close homology among some taxa, it is possible to design and implement eDNA markers with high discriminatory power for key continental terrestrial fauna incorporating both marsupials and eutherian mammals. Our approach can be implemented in other parts of the world by targeting appropriate fauna assemblage in the development of the mini-barcode.

## Data Description

We identified the 12S rRNA gene as a target for development of a mini-barcode marker. We developed a reference DNA database for this gene, including 174 sequences from 24 genera and 41 mammal species. Sequences were obtained from GenBank, with additional targeted sequencing conducted for target species under-represented in GenBank. Sequences were aligned, trimmed to 901 bp, and are provided here in FASTA format (Additional file 1) with additional information on sample and sequence origins in .csv format (Additional file 2).

We[Table tbl1] used the R package SPIDER [47] to conduct a sliding window analysis [2] to identify a short diagnostic region of the 12S rRNA gene suitable for use as a mini-barcode marker. R code for this analysis is provided in text format (Additional file 3).

Following design of the *AusPreda_12S* primers, we conducted bioinformatic and laboratory evaluations of the sensitivity and specificity of the mini-barcode. We created 2 modified versions of our reference 12S rRNA database, trimmed to include only the 178 bp flanked by the mini-barcode *AusPreda_12S* primers. The “FULL” database included all 174 sequences from the original database, while the “UNIQUE” database included a subset of 44 sequences, where singleton species (species represented by only 1 haplotype) were removed and where each remaining haplotype was represented by only a single sequence. These 2 databases are provided here in FASTA format (Additional files 4 and 5). We used the R package SPIDER to conduct genetic distance-based evaluations of the *AusPreda_12S* primers to identify the risks of incorrect or ambiguous species identifications based on this sequence. R code for these analyses is provided in text format (Additional file 6), and detailed results are provided in .csv format (Additional file 7).

We conducted polymerase chain reactions (PCRs) to evaluate amplification success using the *AusPreda_12S* primers on tissue samples from a range of mammal species. Details of samples used are provided in .csv format (Additional file 8). We also tested amplification success from known-origin scats collected from 6 different predator species. All PCR products successfully amplified from scats were sequenced to confirm predator of origin: resulting sequences are provided here in FASTA format (Additional file 9).

## Results

### Development of a new mammal mini-barcode

We selected the 12S rRNA gene as a promising candidate marker for development of a mini-barcode and developed a 12S rRNA reference sequence database for Australian mammals comprising 174 sequences. Within the 12S rRNA gene, we identified a 178-bp diagnostic mini-barcode region that displayed high levels of inter-specific variation. Within this region, the proportion of 0 non-conspecific K2P distances was equal to 0 for windows of 175 bp in length, and the number of diagnostic nucleotides per window was high. We identified 2 potential primer sites with high proportions of 0 non-conspecific K2P distances (>0.8) and low numbers of diagnostic nucleotides (0–1 nucleotides per 20-bp window). We designed 2 conserved primers, *AusPreda_12SF* and *AusPreda_12SR*, to amplify this mini-barcode from a range of mammal species. The final PCR product was 218 bp in length, including the primers.

### Bioinformatic evaluation of the mini-barcode

We used 3 different genetic distance-based analyses to estimate the risks of species mis-identification when using our *AusPreda_12S* primers on samples of unknown origin (Table 1; Additional file 7). These analyses used versions of the 12S rRNA reference sequence database, trimmed to include only the 178-bp mini-barcode region (Additional files 4 and 5). A *nearNeighbour* analysis of all sequences (the “FULL” database) correctly identified 155 sequences and incorrectly identified 19 sequences. All incorrectly identified sequences except 1 western quoll (*D. geoffroii*) from GenBank originated from species for which only a single reference sequence was available (i.e., singleton species), and thus the nearest neighbour was automatically another species. In most cases, this nearest neighbour was a member of the same genus. For example, the nearest neighbour of the only bronze quoll (*D. spartacus*) sequence available was from the western quoll (*D. geoffroii*). This close genetic similarity has also been shown by Woolley et al*.* [48]. The western quoll incorrectly identified with the nearest neighbour analyses was closely related to the bronze quoll, which can indicate that this particular western quoll sequence from GenBank (KJ780027) was possibly mis-identified. Further analyses using a database including only unique haplotypes, from which singleton species were excluded (the “UNIQUE” database), identified correctly all 44 sequences.

**Table 1: tbl1:** Summary of results of genetic distance-based evaluations of the *AusPreda_12S* mini-barcode

	FULL (1% threshold)				UNIQUE (3.5% threshold)			
	Correct/true	Incorrect/false	Ambiguous	No ID	Correct/true	Incorrect/false	Ambiguous	No ID
*Nearest neighbour*	155	19	–	–	44	0	–	–
*Best close match*	147	3	0	24	42	0	0	2
*Thresh ID*	142	3	5	24	42	0	0	2

	FULL (3.5% threshold)			
	Correct/true	Incorrect/false	Ambiguous	No ID
*Best close match*	152	6	0	16
*Thresh ID*	141	5	12	16

Summary of results of genetic distance-based evaluations of the *AusPreda_12S* mini-barcode conducted using the R package SPIDER to analyse the “FULL” (at 1% and 3.5% thresholds) and “UNIQUE” (at 3.5% threshold) reference sequence databases. The thresholds were calculated based on the minimum cumulative error (Additional file 6), and the 3.5% threshold for the “FULL” database allows for comparison between the 2 databases. The specified genetic distance thresholds were used for the *BestCloseMatch* and *ThreshID* analyses.

**Figure 1: fig1:**
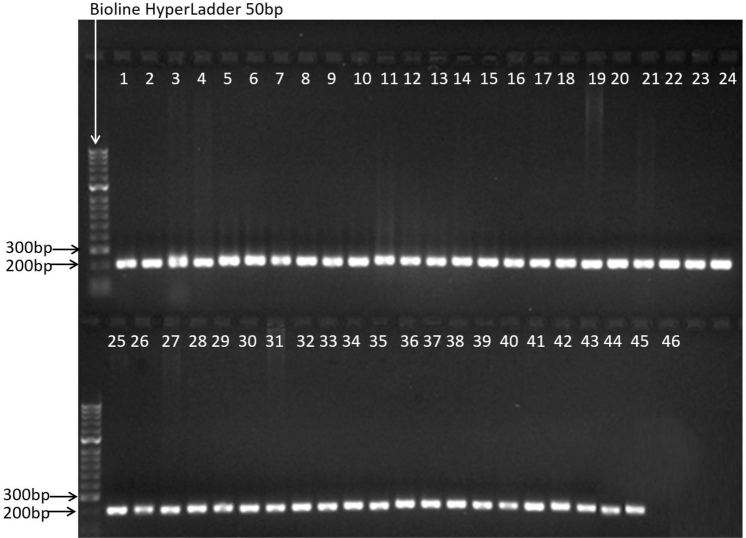
Gel showing amplification success from 45 known tissue samples representing 40 species, using the *AusPreda_12S* mini-barcode primers developed in this study, and a PCR negative. The expected amplicon size is 218 bp. Samples are grouped by species as follows: lanes 1 and 2: *Felis catus*; 3: *Canis lupus familiaris*; 4: *Canis lupus dingo*; 5 and 6: *Dasyurus viverrinus*; 7 and 8: *Dasyurus maculatus*; 9 and 10: *Vulpes vulpes*; 11 and 12: *Sarcophilus harrisii*; 13: *Oryctolagus cuniculus*; 14: *Lepus capensis*; 15: *Bos Taurus*; 16: *Ornithorhyncus anatinus*; 17: *Trichosorus vulpecula*; 18: *Petaurus breviceps*; 19: *Tachyglossus aculeatus*; 20: *Potorous tridactylus*; 21: *Bettongia gaimardi*; 22: *Dactylopsila trivirgata*; 23: *Burramys parvus*; 24: *Macropus rufogriseus*; 25: *Thylogale billardierii*; 26: *Pseudomys gracilacaudatus*; 27: *Pseudocheirus peregrinus*; 28: *Antechinus minimus*; 29: *Tiliqua nigrolutea*; 30*: Vombatus ursinus*; 31: *Isoodon obesulus*; 32: *Macropus giganteus*; 33: *Parameles gunnii*; 34: *Sminthopsis leucopus*; 35: *Mus musculus*; 36: *Planigale gilesi*; 37: *Rattus lutreolus velutinus*; 38: *Phascogale tapoatafa*; 39: *Hydromys chrysogaster*; 40: *Macropus rufus*; 41: *Vicugna pacos*; 42: *Dasyurus hallucatus*; 43: *Lathamus discolour*; 44: *Geocrinia laevis*; 45: *Dasyurus geoffroii*; 46: PCR negative.

*BestCloseMatch* and *ThreshID* analyses, which both assume that sequences from a single species fall within a specified genetic distance threshold, correctly identified 147 and 142 sequences, respectively, in the “FULL” database using the 1% threshold given by the minimum cumulative error. Three sequences were incorrectly identified in both analyses: *Dasyurus spartacus* (AF009892), *Pseudantechinus macdonnellensis* (EU086642), and *Pseudantechinus roryi* (EU086650), each representing singleton species and falling within the 1% genetic distance threshold of a congeneric species, enabling them to be mistaken for their close relatives. Five *D. geoffroii* sequences[Fig fig1] were correctly identified using *BestCloseMatch* but were ambiguously identified in the *ThreshID* analysis because of a close similarity (within the 1% genetic distance threshold) with the single *D. spartacus* sequence*.* A further 24 sequences could not be identified in either analysis because all other sequences within the reference database were more than 1% different. The majority of these sequences were from singletons, but a more relaxed genetic distance threshold (2–5%) identified them correctly. *BestCloseMatch* and *ThreshID* analyses of the “UNIQUE” database identified correctly 42 of 44 sequences, but the 2 remaining sequences, both from *Dasycercus cristicauda*, could not be identified (Table 1; details of results: Additional file 7)*.* As noted previously, these sequences would have been correctly identified if a genetic distance threshold of 5% was used. This represents a high level of divergence between 2 conspecific sequences, but as both of these sequences were obtained from GenBank, and the origin of 1 of the samples is unknown, we cannot rule out sample misidentification or sequencing error in this instance.

Using a 3.5% genetic threshold for the “FULL” database to allow for comparison with the results obtained with the “UNIQUE” database correctly identified more sequences with the *BestCloseMatch* analysis, which was to be expected using a more relaxed genetic threshold, allowing for more mismatches among sequences. Nevertheless, 6 sequences previously resulting in a “No ID” match became correctly identified and 2 became incorrectly identified. The western quoll (KJ780027) became incorrectly identified using a higher threshold, which, once again, led us to believe that this sequence from GenBank was incorrectly identified to start with. Comparing the *ThreshID* results with the more conservative approach used with the 1% threshold, 5 sequences that were previously correctly identified became ambiguous, and from 8 sequences resulting in a “No ID” match, 4 became correctly identified, 2 became incorrectly identified, and 2 had an ambiguous identification.

### Evaluation of the amplification success and sensitivity of the *AusPreda_12S* primers

Our mini-barcode was successfully amplified from all 45 tissue samples tested, including samples from a wide taxonomic range of Australian mammals (40 species), as well as a reptile, an amphibian, and a bird (Fig. 1; Additional file 8). This demonstrates the broad applicability of the primers across the mammalian taxa and their potential applicability to other vertebrate classes. Because we aimed to target both marsupial and eutherian mammals, we were unable to identify a mini-barcode that amplified only the 6 target species.

We also successfully amplified our mini-barcode from a wide range of input template DNA concentrations. We set up serial dilutions of DNA from 6 predator species. Amplification was successful for all 3 qPCR replicates from all 6 species for all dilutions from 9 ng/μl to 9 pg/μl inclusive, demonstrating that the primers can amplify from low-quantity DNA. Amplification success was less consistent at the highest and lowest DNA concentrations, estimated at 90 ng/μl, 0.9 pg/μl, and 0.09 pg/μl (Table 2), indicating that reliability of predator detection from DNA below 9 pg/μl may be poor. Failure to amplify from highly concentrated DNA, despite successful amplification from dilutions of the same DNA extracts, may reflect the presence of PCR inhibitors in these extracts, which were obtained from museum and roadkill specimens.

**Table 2:** Results of qPCR tests conducted to evaluate amplification success of the *AusPreda_*12S mini-barcode from low-template DNA; 6 DNA samples were serially diluted, with amplification success determined by comparison of CT values^a^ for 3 replicates of each dilution

Species	Dilution	Replicate 1	Replicate 2	Replicate 3	CT mean^b^
Cat N22b	1 in 10 (9 ng/μl)	12.444	14.281	13.373	13.366
	1 in 100 (0.9 ng/μl)	16.346^c^	13.399	13.368	13.384
	1 in 1000 (0.09 ng/μl)	19.252	23.382	23.994	22.209
	1 in 10 000 (9 pg/μl)	31.252	27.486	27.604	28.781
	1 in 100 000 (0.9 pg/μl)	31.483	31.476	29.386	30.782
	1 in 1 000 000 (0.09 pg/μl)	Undetermined	Undetermined	Undetermined	–
Dingo AA15020	1 in 10 (9 ng/μl)	14.303	13.019	15.363	14.228
	1 in 100 (0.9 ng/μl)	15.879	16.791	16.623	16.431
	1 in 1000 (0.09 ng/μl)	19.719	19.237	17.424	18.793
	1 in 10 000 (9 pg/μl)	22.652	24.957	25.196	24.268
	1 in 100 000 (0.9 pg/μl)	Undetermined	Undetermined	Undetermined	–
	1 in 1 000 000 (0.09 pg/μl)	Undetermined	Undetermined	Undetermined	–
Eastern quoll UC1214	1 in 10 (9 ng/μl)	14.128	13.509	13.449	13.695
	1 in 100 (0.9 ng/μl)	17.267	20.866^c^	17.235	17.251
	1 in 1000 (0.09 ng/μl)	17.662	21.523	21.385	20.190
	1 in 10 000 (9 pg/μl)	24.346	26.474	25.653	25.491
	1 in 100 000 (0.9 pg/μl)	Undetermined	Undetermined	34.570	34.570
	1 in 1 000 000 (0.09 pg/μl)	Undetermined	Undetermined	Undetermined	–
Spotted-tailed quoll A3395	1 in 10 (9 ng/μl)	13.460	13.928	14.048	13.812
	1 in 100 (0.9 ng/μl)	17.517	16.447	18.653	17.539
	1 in 1000 (0.09 ng/μl)	20.374	19.540	17.003	18.972
	1 in 10 000 (9 pg/μl)	27.511	25.453	23.851	25.605
	1 in 100 000 (0.9 pg/μl)	30.158	30.132	25.107	28.466
	1 in 1 000 000 (0.09 pg/μl)	Undetermined	35.172	Undetermined	35.172
Red fox UC0401	1 in 10 (9 ng/μl)	15.547	15.528	14.628	15.234
	1 in 100 (0.9 ng/μl)	19.566	17.524	16.860	17.983
	1 in 1000 (0.09 ng/μl)	21.915	22.827	22.360	22.367
	1 in 10 000 (9 pg/μl)	26.672	25.460	25.508	25.880
	1 in 100 000 (0.9 pg/μl)	31.672	30.914	28.863	30.483
	1 in 1 000 000 (0.09 pg/μl)	Undetermined	31.601	Undetermined	31.601
Tasmanian devil A3357	1 in 10 (9 ng/μl)	15.502	16.810^c^	14.536	15.019
	1 in 100 (0.9 ng/μl)	19.736	18.729	19.702	19.389
	1 in 1000 (0.09 ng/μl)	23.517	22.999	21.591	22.702
	1 in 10 000 (9 pg/μl)	27.216	28.006	24.130	26.451
	1 in 100 000 (0.9 pg/μl)	30.876	30.734	28.977	30.196
	1 in 1 000 000 (0.09 pg/μl)	32.534	Undetermined	Undetermined	32.534

^a^Numbers represent observed CT values for each replicate qPCR of a series of DNA dilutions. The CT value represents the number of cycles required for the fluorescent signal of a qPCR machine to cross the predetermined threshold, here set at 5000 ΔRn.

^b^Undetermined results were excluded when calculating mean CT.

^c^Where the qPCR traces were of an irregular shape (3 replicates), the replicate was excluded when calculating mean CT.

### Evaluation of amplification success from trace samples using known-origin scats

We tested the ability of the *AusPreda_12S* primers to correctly identify known predators by analysing scats from captive animals. A total of 57 scats were tested, and amplified product was obtained from 53 samples. We obtained good-quality DNA sequences, ranging from 116 bp to 182 bp in length, from 49 (92%) of these 53 scats (Additional file 9). The species of origin was correctly identified for all 49 samples, with scat DNA sequences matched to appropriate GenBank reference sequences with 97–100% sequence identity (Table 3).

**Table 3:** PCR and DNA sequencing results from 57 known-origin scat samples screened using the *AusPreda_12S* mini-barcode

Sample	Scientific name	Common name	Amplified	Sequenced	Closest sequence match using BLAST	% ID^a^	e value^b^
100111–27	*Canis lupus familiaris*	Dog	Y	Y	Dog	99.4	1.55E-84
120111–02	*Canis lupus familiaris*	Dog	Y	Y	Dog	100	6.52E-78
121010–11	*Canis lupus familiaris*	Dog	Y	Y	Dog	99.4	1.22E-85
121010–16	*Canis lupus familiaris*	Dog	Y	Y	Dog	98.4	2.08E-83
121010–17	*Canis lupus familiaris*	Dog	Y	Y	Dog	99.4	1.98E-83
121010–30	*Canis lupus familiaris*	Dog	Y	Y	Dog	99.4	5.54E-84
121010–52	*Canis lupus familiaris*	Dog	Y	N	NA	NA	NA
121010–53	*Canis lupus familiaris*	Dog	Y	Y	Dog	98.9	2.60E-82
121010–54	*Canis lupus familiaris*	Dog	Y	Y	Dog	99.4	1.22E-85
121010–56	*Canis lupus familiaris*	Dog	Y	Y	Dog	98.9	7.22E-83
121110–55	*Canis lupus familiaris*	Dog	Y	Y	Dog	99.4	5.54E-84
170211–12	*Canis lupus familiaris*	Dog	N	NA	NA	NA	NA
041110–66	*Dasyurus maculatus*	Spotted-tailed quoll	Y	Y	Spotted-tailed quoll	98.4	2.08E-83
101110–9	*Dasyurus maculatus*	Spotted-tailed quoll	Y	Y	Spotted-tailed quoll	98.2	2.33E-72
170211–25	*Dasyurus maculatus*	Spotted-tailed quoll	Y	Y	Spotted-tailed quoll	99.4	1.55E-84
041110–01	*Dasyurus viverrinus*	Eastern quoll	Y	Y	Eastern quoll	99.4	2.25E-72
041110–04	*Dasyurus viverrinus*	Eastern quoll	Y	Y	Eastern quoll	100	2.05E-88
041110–07	*Dasyurus viverrinus*	Eastern quoll	Y	Y	Eastern quoll	100	4.80E-74
041110–15	*Dasyurus viverrinus*	Eastern quoll	Y	Y	Eastern quoll	100	1.01E-54
041110–74	*Dasyurus viverrinus*	Eastern quoll	Y	Y	Eastern quoll	100	1.19E-85
041110–80	*Dasyurus viverrinus*	Eastern quoll	Y	Y	Eastern quoll	100	9.34E-87
100111–05	*Dasyurus viverrinus*	Eastern quoll	Y	Y	Eastern quoll	100	3.34E-86
100111–31	*Dasyurus viverrinus*	Eastern quoll	Y	Y	Eastern quoll	100	3.34E-86
120111–32	*Dasyurus viverrinus*	Eastern quoll	N	NA	NA	NA	NA
120111–33	*Dasyurus viverrinus*	Eastern quoll	Y	Y	Eastern quoll	100	2.61E-87
170211–14	*Dasyurus viverrinus*	Eastern quoll	Y	Y	Eastern quoll	100	2.61E-87
100111–04	*Felis catus*	Feral cat	Y	Y	Feral cat	100	1.54E-79
120111–10	*Felis catus*	Feral cat	Y	Y	Feral cat	100	1.56E-79
120111–12	*Felis catus*	Feral cat	Y	Y	Feral cat	100	1.58E-79
120111–31	*Felis catus*	Feral cat	Y	N	NA	NA	NA
170211–13	*Felis catus*	Feral cat	Y	Y	Feral cat	99.2	3.36E-60
170211–21	*Felis catus*	Feral cat	Y	Y	Feral cat	100	1.61E-79
170211–22	*Felis catus*	Feral cat	Y	Y	Feral cat	100	1.55E-79
041110–42	*Sarcophilus harrisii*	Tasmanian devil	Y	Y	Tasmanian devil	100	4.02E-80
041110–47	*Sarcophilus harrisii*	Tasmanian devil	Y	Y	Tasmanian devil	100	9.34E-87
041110–48	*Sarcophilus harrisii*	Tasmanian devil	Y	Y	Tasmanian devil	100	2.61E-87
041110–53	*Sarcophilus harrisii*	Tasmanian devil	Y	Y	Tasmanian devil	100	2.47E-82
041110–59	*Sarcophilus harrisii*	Tasmanian devil	Y	Y	Tasmanian devil	100	7.32E-88
121010–06	*Sarcophilus harrisii*	Tasmanian devil	Y	Y	Tasmanian devil	100	4.02E-80
121010–22	*Sarcophilus harrisii*	Tasmanian devil	Y	Y	Tasmanian devil	99.4	5.58E-84
200910–24	*Sarcophilus harrisii*	Tasmanian devil	Y	Y	Tasmanian devil	100	9.34E-87
200910–25	*Sarcophilus harrisii*	Tasmanian devil	Y	Y	Tasmanian devil	100	2.61E-87
080211–04	*Vulpes vulpes*	Red fox	Y	Y	Red fox	99.4	1.22E-85
080211–05	*Vulpes vulpes*	Red fox	Y	Y	Red fox	99.4	5.54E-84
080211–06	*Vulpes vulpes*	Red fox	Y	N	NA	NA	NA
080211–07	*Vulpes vulpes*	Red fox	Y	Y	Red fox	97.2	9.35E-61
080211–08	*Vulpes vulpes*	Red fox	Y	Y	Red fox	99.4	5.54E-84
080211–09	*Vulpes vulpes*	Red fox	N	NA	NA	NA	NA
080211–10	*Vulpes vulpes*	Red fox	Y	Y	Red fox	100	6.52E-78
080211–11	*Vulpes vulpes*	Red fox	Y	Y	Red fox	98.9	5.66E-84
080211–12	*Vulpes vulpes*	Red fox	Y	N	NA	NA	NA
080211–13	*Vulpes vulpes*	Red fox	Y	Y	Red fox	98.8	3.99E-75
080211–14	*Vulpes vulpes*	Red fox	Y	Y	Red fox	100	6.52E-78
080211–15	*Vulpes vulpes*	Red fox	Y	Y	Red fox	99.1	2.63E-50
080211–16	*Vulpes vulpes*	Red fox	Y	Y	Red fox	100	6.52E-78
080211–17	*Vulpes vulpes*	Red fox	N	NA	NA	NA	NA
080211–18	*Vulpes vulpes*	Red fox	Y	Y	Red fox	97.8	1.23E-80

^a^% ID is the percentage pairwise identity between the query sequence and the matching sequence identified using BLAST.

^b^The e-value represents the number of BLAST hits expected by chance. The lower the e-value is, the better.

## Discussion

Non-invasive environmental DNA-based methods can provide a novel approach to the detection of cryptic animals in large-scale surveys [49], with applications to wildlife management. Such DNA approaches can make important contributions to the ability to detect incursions or monitor established invasive species [41, 50, 51] or to detecting very rare or declining species of conservation significance [8, 52].

Here, we report a PCR-based mini-barcode test for medium-large Australian mammalian predators. This test can amplify DNA from and discriminate among the 4 quoll species found in Australia, as well as the Tasmanian devil (the only other extant large marsupial predator) and introduced mammal carnivores, with a high level of accuracy. We expect that these primers will also amplify DNA from both species of New Guinean quoll. Previous studies aimed at identifying species from scats or hairs have applied barcoding methods to detect individual species across multiple time points (examples in [53, 54]). Here we have shown that it is also possible to identify multiple species by implementing a single DNA test using a straightforward PCR and Sanger sequencing approach. All clear sequences obtained from 49 scats of 6 target predator species were correctly identified to the species level. In the small number of cases where a clear sequence was not obtained from a scat, we found that the sequences obtained were mixed, probably arising from the amplification of 2 or more species in the same sample. This could arise from cross-contamination among samples but is more likely the result of the amplification of prey DNA present in the scat [14, 55]. We have previously observed this phenomenon when using a single species test to detect fox DNA, where rabbit or hare DNA were sometimes erroneously amplified [37]. This demonstrates the need to account for the history of samples analysed (how they were obtained, how fresh they were upon collection, and how samples and DNA extracts were stored) and the importance of a DNA sequencing step in any of these analyses to enable recognition of non-specific PCR amplification. In practice, mixed sequences cannot be used to identify the predator with confidence, and therefore such samples must be excluded from analysis. In addition to successful amplification of scat DNA, we demonstrate that our mini-barcode primers can successfully amplify low-template DNA (at least as low as 0.9 pg/μl) from museum samples. This provides further evidence of the utility of this marker for application to eDNA studies.

Whilst DNA metabarcoding may more clearly determine which species are represented in mixed samples, metabarcoding methods are relatively costly and require more specialist equipment, which may not be available to many wildlife managers. In this study, PCR and Sanger sequencing reliably identified the predator of origin for 86% of scat samples, which is likely to be sufficient for many management applications and is a higher success rate than has been reported for several other faecal DNA studies (e.g., [41], where 79% of sequences were amplified using a 134-bp fragment, and [56], where <70% of sequences were amplified using regions ranging from 243 bp to 708 bp according to target taxon). Using our mini-barcode, DNA can be screened for the presence of multiple Australian predator species in a single and inexpensive test, without the need to develop and apply a set of species-specific primers for each predator of interest. We provide a non-invasive instrument with potential utility for scientists or managers working with endangered or invasive Australian predators, but a similar approach could be used to target predator assemblages in other regions.

The bioinformatic evaluation of our mini-barcode shows that this marker can reliably discriminate among the 8 target predator species (eastern, western, northern, and spotted-tail quolls, Tasmanian devils, cats, dogs, and foxes) in Australia. The close genetic similarity between the bronze quoll (from New Guinea) and the western quoll (from Australia), described above and supported by Woolley, Krajewski, and Westerman [48], may pose some problems for reliable species identification from unknown samples, but the different geographic distributions of these 2 species will likely provide a clear identification in most cases. The most appropriate threshold to be used will depend on the management context and the relative importance of false-positive identifications, but in most cases, an ambiguous or “No ID” identification would be a better result for a sample than to result in a correct identification when this is erroneous.

Further development of our reference database, to include additional *D. albopunctatus* and *D. spartacus* sequences, will be required to better understand the utility of this test for identification of specimens to the species level in New Guinea. Likewise, a better reference database would improve the relevance of this DNA test for application to historic samples. Sequences from the extinct thylacine could be clearly identified in our initial analyses, but this species could not be included in the UNIQUE database for further bioinformatic analysis because only 1 12S rRNA haplotype was available. Finally, because we are working with mitochondrial DNA, which is maternally inherited, we cannot currently use this test to distinguish between dogs and dingos, in part because of the prevalence of hybrids in many wild populations [57, 58].

### Considerations when working with scats

One important consideration for future studies using the *AusPreda_12S* primers is the need to understand the ecological role of the species from which eDNA is detected. Typically, predator DNA is the most abundant in scats, owing to the release of epithelial cells during defecation [59–61]. However, because there are multiple potential sources of DNA in scat samples, it is also possible that these primers will amplify DNA from prey species. In some cases, this will be obvious, e.g., where the scats of the prey species detected are clearly morphologically different from carnivore scats. However, other results may be more difficult to interpret, e.g., where mixed sequences, representing 2 different predator species that could potentially predate upon one another, are obtained from the same sample.

### Conservation implications

The *AusPreda_12S* primers provide an opportunity to enhance monitoring of predators across Australia for conservation purposes [62]. For example, western quolls were successfully re-established in Western Australia in 1987 after a recovery plan was implemented over 13 years, in areas previously baited with 1080 to remove introduced species [63]. Western quolls from Western Australia were also re-introduced to the Flinders Ranges in South Australia in 2014, and that population is now breeding in the wild, with more than 60 young born since their relocation [64, 65]. Eastern quolls were re-introduced from Tasmania to Mulligans Flat Woodland Sanctuary, in the Australian Capital Territory, in early 2016 [66]. There are also proposals to reintroduce devils to south-eastern mainland Australia to reduce the negative impact that dingo control has on small mammals through mesopredator release [67–70]. The development of this mini-barcode now provides a new tool with which to monitor these re-introduced species, and the non-native predators that threaten them, from non-invasive samples.

### Future work

In the future, this predator identification tool may be used to model the distribution of predators in Tasmania or mainland Australia, supplementing more traditional data obtained from live trapping and sightings. It is now possible to reliably detect a predator of interest from non-invasive samples. Using the *AusPreda_12S* primers in an initial sample screening step may provide further opportunities to study the diets of each specific predator by identifying samples to include in targeted metabarcoding studies. This test could also be more broadly useful, with potential application to detection and monitoring of the 2 New Guinean quoll species.

## Methods

### Selection of a candidate marker gene

We compiled initial reference databases for 3 mitochondrial genes, 12S rRNA, 16S rRNA, and ND2, all of which have proven useful for species detection in other studies [61, 71–74]. These databases used sequences collected mainly from GenBank (GenBank, RRID:SCR_002760) [75, 76].

We used the R package SPIDER to identify potential mini-barcodes from these initial reference databases. Our criteria were to identify regions of between 100 and 200 bp in length (the maximum that can be reasonably amplified from many eDNA samples) that displayed high levels of inter-specific variation within the region and that were flanked by primer sites that were well conserved across all taxa, but particularly across our 6 key Tasmanian target species. For each gene, we conducted a sliding window analysis with window sizes of 100, 125, 150, and 175 bp to identify potential mini-barcodes. For each window, we evaluated the number of diagnostic nucleotides per window and the proportion of 0 non-conspecific K2P distances to identify regions with high inter-specific variation that may be used to discriminate among species. Subsequently, we used further sliding window analyses to identify conserved primer sites adjacent to candidate mini-barcode regions. We used window sizes of 20, 25, and 30 bp to identify potential sites for primer development. Of these, a window size of 20 gave the best results, so we adopted 20 bp as the standard primer length.

We were not able to identify any candidate mini-barcode markers that met all of our criteria from the 16S rRNA and ND2 genes, so all subsequent work was focused on the 12S rRNA gene.

### Development of a reference database for the 12S rRNA gene

We constructed a reference database for the 12S rRNA gene. This included representatives of native and introduced Tasmanian mammal predators and their potential prey species, their mainland Australian relatives, livestock, and other introduced species (i.e., goat, sheep, horse, wild boar, cow, and fallow deer) and humans. Importantly, all 6 recognized quoll species (4 Australian and 2 New Guinean) were represented (Additional files 1 and 2). The final reference database consisted of 174 sequences, representing 41 species from 24 genera. We obtained the majority of sequences from GenBank, but we generated additional sequences from a selection of species that were under-represented in the public database. DNA was extracted from tissue samples from museum specimens, road-killed animals, and western quoll tissues collected during a reintroduction program in the Flinders Ranges (South Australia) involving quolls of Western Australian origin [77]. We used a salting out method [78], with minor modifications as follows. Our lysis buffer included 10% Sodium dodecyl sulfate (SDS) (Sigma-Aldrich), and tissues were digested in a thermomixer for 3 hours at 56°C with mixing at 500 rpm. DNA pellets were air dried for 30–60 minutes and re-suspended in 50 μl of ddH_2_O. Genomic DNA extracts were quantified using a Nanodrop ND1000 spectrophotometer (Thermo Fischer Scientific), and samples were diluted with ddH2O to a final concentration of *ca* 40 ng/μl. The entire 12S gene region was amplified by PCR using primers 12C and 12gg (Table 4). PCRs of 25 μl final volume contained 0.4 μM of each primer, ×1 MyTaq^TM^ red mix (Bioline) and *ca* 3.2 ng/μl of genomic DNA. Cycling conditions were: 95°C for 2 minutes; 10 cycles of 95°C for 20 seconds, a touchdown from 60–50°C for 20 seconds, and 72°C for 1 minute; then 35 cycles of 95°C for 20 seconds, 50°C for 20 seconds, and 72°C for 1 minute; followed by a final extension at 72°C for 4 minutes. PCR products were visualized on a 1.7% Tris/Borate/EDTA (TBE) agarose gel (Agarose I: Amresco, Solon, OH, USA) run for 40 minutes at 90 V. Hyperladder 50 bp (Bioline, Australia) was included to serve as a size reference. Amplicons were cleaned using Diffinity rapid tips (Scientific Specialties, Inc., CA, USA) and prepared for sequencing following protocols recommended by the Biomolecular Resource Facility (Australian National University) before being sequenced in both directions on a 96 capillary 3730 DNA Analyzer (Applied Biosystems). Forward and reverse sequences for each sample were manually checked, trimmed of primer sequences and low-quality bases at the 3΄ ends, and aligned using Geneious 8.1.7 (Biomatters, Auckland, New Zealand) [79]. The final alignment was 901 bp in length.

**Table 4:** PCR primers used in this study

Marker	Sequence (5΄ – 3΄)	Amplicon length	Reference
*12C* & *12GG*	12C: AAAGCAAARCACTGAAAATG	1061 bp	[80]
	12GG: TRGGTGTARGCTRRRTGCTTT		
*AusPreda_12S*	AusPreda_12SF: CCAGCCACCGCGGTCATACG	218 bp	This study
	AusPreda_12SR: GCATAGTGGGGTCTCTAATC		

### Development of primers for the mini-barcode

**Figure 2:**
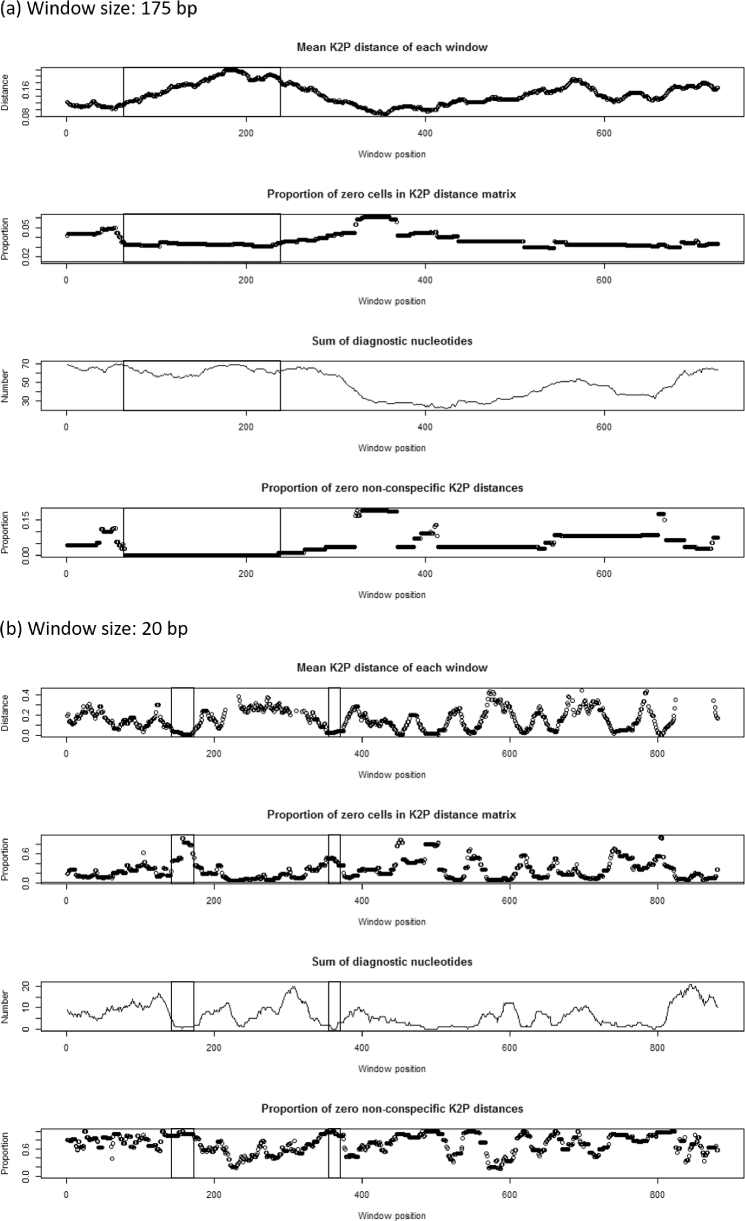
Results of the sliding window analysis conducted using the R package SPIDER for the 12S rRNA gene using window sizes of **(a)** 175 bp and **(b)** 20 bp to identify candidate mini-barcode regions and conserved primer sites, respectively. For all panels, the x-axes represent the position of each window within the sequence alignment, with each data point marking the position of the first nucleotide of 1 window. The first (top) panels display the mean K2P distances (a measure of genetic differentiation among species, where a value of 0 means that sequences are identical) calculated for each window, with K2P values represented on the y-axes. The second panels represent the proportion of 0 cells in the K2P distance matrix. A high proportion of inter-specific genetic distances that are equal to 0 indicates sequences that are highly conserved among species. The third panels display the number of nucleotides that are diagnostic among species within each window. The fourth (lowest) panels indicate the proportion of 0 non-conspecific K2P distances within each window. When this value is 0, it indicates that the sequence region has high potential to discriminate among species. The area boxed within each panel denotes (a) the regions containing the first bases where a mini-barcode of *ca* 175 bp can be developed and (b) the regions containing the first bases where conserved primer sites can be developed.

A sliding window analysis of our 12S rRNA reference database, using the R package SPIDER [47], identified a candidate mini-barcode of 344 bp in length. The proportion of 0 non-conspecific K2P distances was equal to 0 for bases 66 to 410 of our alignment, using a sliding window analysis with 175 bp windows, and each window included high numbers of diagnostic nucleotides (51–69 per window). Within this candidate mini-barcode, a sliding window analysis using 20 bp windows identified 2 short, highly conserved regions suitable for primer design (Fig. 2; Additional file 3). These potential primer sites had a high proportion of 0 non-conspecific K2P distances (>0.8) and low numbers of diagnostic nucleotides (0–1 per window). Within these regions, we manually designed the primers *AusPreda_12SF* (5΄-CCAGCCACCGCGGTCATACG-3΄) and *AusPreda_12SR* (5΄-GCATAGTGGGGTCTCTAATC-3΄) (Table 4). These primers flank a region of high inter-specific variation and amplify a product of 218 bp in length (178 bp excluding primers).

### Bioinformatic evaluation of the mini-barcode

We used additional functions of the R package SPIDER to estimate the risks of species mis-identification when using our *AusPreda_12S* primers on samples of unknown origin. These analyses were conducted using 2 versions of our 12S reference database, trimmed to include only the 178 bp of sequences flanked by the *AusPreda_12S* primers. The “FULL” database included all 174 sequences present in the original database (Additional file 4). The “UNIQUE” database was a subset of the “FULL” database in which each haplotype was represented by only a single sequence and in which singleton species (species represented by only 1 haplotype) were removed. This included 44 sequences, representing 16 species from 12 genera (Additional file 5).

Pairwise genetic distance was calculated for each pair of sequences using the “raw” model. We conducted bioinformatic analyses using the *nearNeighbour*, *BestCloseMatch*, and *ThreshID* functions to identify the taxa most likely to be misidentified or ambiguously identified using our primers. R code for these analyses is provided in Additional file 6. The *nearNeighbour* function determines, for each sequence in the reference database, whether the most closely related sequence originates from a conspecific, with 2 outcomes possible: “true” or “false.” A genetic distance threshold must be specified for the *BestCloseMatch* and *ThreshID* functions to account for intra-specific variation. We estimated the most appropriate genetic thresholds to use for the “UNIQUE” and “FULL” databases to be 3.5% and 1%, respectively, based on the thresholds with the lowest cumulative error. The *BestCloseMatch* analysis identified the most closely related sequence, within the specified genetic distance threshold, and its species of origin for each query sequence. The *ThreshID* analysis extended this to consider species of origin for all sequences within the genetic distance threshold. These analyses had 4 possible outcomes: “correct,” “incorrect,” “ambiguous,” and “no identification” [47]. The “FULL” database was also analysed, with a 3.5% genetic threshold to allow for comparison with the results of the “UNIQUE” database.

### Evaluation of the amplification success and sensitivity of the *AusPreda_12S* primers

We screened a panel of DNA samples from 45 specimens representing 40 species (Additional file 8) to evaluate the amplification success of the *AusPreda_12S* primers. DNA was extracted from tissue samples as described above and amplified with the *AusPreda_12S* primers using the same cycling conditions as for the 12C and 12gg primers above, with PCR products visualized on a 1.7% TBE agarose gel to determine amplification success (Fig. 1).

To test the sensitivity of our primers to detect low template DNA samples, we set up serial dilutions of 6 DNA extracts originating from museum samples, representing each of the 6 mammal predators that might be detected in Tasmania (Tasmanian devil, eastern quoll, spotted-tail quoll, cat, dog, and fox). The DNA concentration of each original DNA extraction was determined using a QuBit Fluorometer and the Qubit dsDNA BR Assay Kit (Thermo Fisher) and diluted with ddH_2_O if necessary to obtain a starting concentration of 90 ng/μl. We then set up a series of 6 ×10 dilutions from each of these “undiluted” (90 ng/μl) samples. For each dilution of each sample, we performed 3 qPCR replicates, each with a total volume of 25 μl including 1X Gold buffer (Applied Biosystems), 2 mM MgCl^2^, 0.4 mg/ml BSA, 0.4 μM of each primer, 0.6 μl SYBR green (1:2000 Life Technologies nucleic acid gel stain), 0.25 mM of each dNTP, 1 unit of AmpliTaq Gold^TM^ (Applied Biosystems), and 2 μl of the appropriate DNA dilution. qPCRs were conducted using the Viia7 Real-Time PCR System (Thermo Fisher Scientific) with an initial step of 95°C for 5 minutes; followed by 40 cycles of 95°C for 30 seconds, 57°C for 30 seconds, and 72°C for 30 seconds. We conducted a comparative cycle threshold (CT) analysis using the ViiA7 software, v. 1.2.4, with a threshold of 5000 ΔRn. For each dilution of each DNA sample, we calculated the mean CT value and the standard deviation across PCR replicates.

### Evaluation of amplification success from trace samples using known-origin scats

We used previously extracted DNA from 57 scats of known origin collected in 2010–2011 from captive animals, including eastern quolls, spotted-tailed quolls, Tasmanian devils, foxes, cats, and dogs. DNA was extracted using a combined chelex (Bio Rad Laboratories, Hercules, CA, USA) and spin column (Mega quick-spin Total Fragment DNA Purification Kit, Intron Biotechnology) methods [81]. We evaluated amplification success from these samples using the *AusPreda_12S* primers by conducting PCRs and visualizing PCR products by gel electrophoresis as described above.

All amplified products were sequenced in both directions using the *AusPreda_12S* primers, following the methods described above for primers 12C and 12gg. Forward and reverse reads were aligned in Geneious 8.1.7 using a global alignment with free end gaps (Geneious alignment) allowing 65% similarity. Primers were trimmed, and a consensus sequence was generated for each sample. Consensus sequences were compared against the GenBank database using nucleotide Basic Local Alignment Search Tool (BLAST; NCBI BLAST, RRID:SCR_004870, MEGABLAST with the “nr” option and a maximum hit of 20) to identify the most likely species of origin.

## Availability of supporting data and material

The datasets and R code associated with this article are provided as supporting information. All DNA sequences generated during this study have been submitted to GenBank: accession numbers KX786294 to KX786344. Details on the method used to evaluate the sensitivity of a mini-barcode can also be found in Protocols.io [82].

Additional file 1: 12S rRNA reference sequence database used for primer design (FASTA format).

Additional file 2: Samples included in the 12S rRNA reference sequence database used for primer design (.csv format).

Additional file 3: R code for sliding windows analysis implemented using SPIDER (text format).

Additional file 4: Reference database used for genetic distance-based evaluation of the *AusPreda_12S* mini-barcode: “FULL” database (FASTA format).

Additional file 5: Reference database used for genetic distance-based evaluation of the *AusPreda_12S* mini-barcode: “UNIQUE” database (FASTA format).

Additional file 6: R code for genetic distance-based evaluation of the *AusPreda_12S* mini-barcode implemented using SPIDER (text format).

Additional file 7: Detailed results of genetic distance-based evaluation of the *AusPreda_12S* mini-barcode (.csv format).

Additional file 8: Samples included in the laboratory evaluation of the *AusPreda_12S* mini-barcode (.csv format).

Additional file 9: Consensus sequences obtained from 53 known-origin scats by amplification with the *AusPreda_12S* mini-barcode (FASTA format).

## Abbreviations

BLAST: Basic Local Alignment Search Tool (tool available through NCBI to compare an unknown sequence to existing sequences in a public database); bp: base pairs (pairs of nucleotides in a DNA or RNA strand); CT value: cycle threshold (the number of cycles required for the fluorescent signal of a qPCR machine to cross the predetermined threshold); eDNA: environmental DNA; mtDNA: mitochondrial DNA; PCR: polymerase chain reaction (a method used to amplify a target DNA or RNA strand); rRNA: ribosomal ribonucleic acid; TBE: Tris/Borate/EDTA (buffer for gel electrophoresis).

## Competing interests

The authors declare that they have no competing interests.

## Funding

This study was funded by the Invasive Animals Cooperative Research Centre as part of project 1.L.21.

## Author contributions

E.M., A.M., and S.S. designed the study. E.M. performed the experiments. E.M. and A.M. analysed the data. E.M. wrote the manuscript, and A.M. and S.S. provided extensive comments. All authors read and approved the final manuscript.

## Supplementary Material

GIGA-D-17-00051_Original-Submission.pdf

GIGA-D-17-00051_Revision-1.pdf

Response-to-Reviewer-Comments_Original-Submission.pdf

Reviewer-1-Report-(Original-Submission).pdf

Reviewer-2-Report-(Original-Submission).pdf

Reviewer-2-Report-(Revision-1).pdf

Additional Files
