# Supplementary material for: A single mini-barcode test to screen for Australian mammalian predators from environmental samples
Source: Gigascience. 2017 Jul 4;6(8):1–13. doi: 10.1093/gigascience/gix052 (PMC5545080; doi:10.1093/gigascience/gix052)
Supplement: GIGA-D-17-00051_Original-Submission.pdf [file gix052_giga-d-17-00051_original-submission.pdf]

GigaScience
Mini-barcode for species identification
--Manuscript Draft--

**Manuscript Number:**	GIGA-D-17-00051
**Full Title:**	Mini-barcode for species identification
**Article Type:**	Research
**Funding Information:**	Invasive Animals Cooperative Research Centre (Project 1.L.21) — Miss Elodie Modave
**Abstract:**	Background: Identification of species from trace samples is now possible through the comparison of diagnostic DNA fragments against reference DNA sequence databases. DNA detection of animals from non-invasive samples, such as predator faeces (scats) that contain traces of DNA from their species of origin, has proved to be a valuable tool for management of elusive wildlife. However, application of this approach can be limited by the availability of appropriate genetic markers. Scat DNA is often degraded, meaning that longer DNA sequences, including standard DNA barcoding markers, are difficult to recover. Instead, targeted short diagnostic markers are required to serve as diagnostic mini-barcodes. The mitochondrial genome is a useful source of such trace DNA markers, because it provides good resolution at species level and occurs in high copy numbers per cell.<br>Results: We developed a mini-barcode, based on a short (178 bp) fragment of the conserved 12S rRNA mitochondrial gene sequence, with the goal of discriminating amongst the scats of large mammalian predators of Australia. We tested the sensitivity and specificity of our primers and can accurately detect and discriminate amongst quolls, cats, dogs, foxes and devils from trace DNA samples.<br>Conclusions: Our approach provides a cost effective, time efficient and non-invasive tool that enables identification of all eight medium-large mammal predators in Australia, including native and introduced species, using a single test. With modification, this approach is likely to be of broad applicability elsewhere.
**Corresponding Author:**	Elodie Modave<br>University of Canberra<br>AUSTRALIA
**Corresponding Author Secondary Information:**	
**Corresponding Author's Institution:**	University of Canberra
**Corresponding Author's Secondary Institution:**	
**First Author:**	Elodie Modave
**First Author Secondary Information:**	
**Order of Authors:**	Elodie Modave<br>Anna Jane MacDonald, BSc, PhD<br>Stephen Donald Sarre, BAppSc, MAppSc, PhD
**Order of Authors Secondary Information:**	
**Opposed Reviewers:**	
**Additional Information:**	
**Question**	**Response**
Are you submitting this manuscript to a special series or article collection?	No
**Experimental design and statistics**	Yes

Powered by Editorial Manager® and ProduXion Manager® from Aries Systems Corporation

Full details of the experimental design and statistical methods used should be given in the Methods section, as detailed in our Minimum Standards Reporting Checklist. Information essential to interpreting the data presented should be made available in the figure legends.<br><br>Have you included all the information requested in your manuscript?	
**Resources**<br><br>A description of all resources used, including antibodies, cell lines, animals and software tools, with enough information to allow them to be uniquely identified, should be included in the Methods section. Authors are strongly encouraged to cite Research Resource Identifiers (RRIDs) for antibodies, model organisms and tools, where possible.<br><br>Have you included the information requested as detailed in our Minimum Standards Reporting Checklist?	Yes
**Availability of data and materials**<br><br>All datasets and code on which the conclusions of the paper rely must be either included in your submission or deposited in publicly available repositories (where available and ethically appropriate), referencing such data using a unique identifier in the references and in the "Availability of Data and Materials" section of your manuscript.<br><br>Have you have met the above requirement as detailed in our Minimum Standards Reporting Checklist?	Yes

Powered by Editorial Manager® and ProduXion Manager® from Aries Systems Corporation

Manuscript

Click here to download Manuscript Mini-barcode for species identification.docx

For submission to GigaScience

Title:

A single mini-barcode test to screen for Australian mammalian predators from environmental samples

Authors:

Elodie Modave[1], Anna J MacDonald[1] and Stephen D Sarre[1]

Address:

[1] Institute for Applied Ecology, University of Canberra, ACT, 2601, Canberra, Australia

Email addresses:

Elodie Modave: Elodie.modave@canberra.edu.au

Anna J MacDonald: Anna.MacDonald@canberra.edu.au

Stephen D Sarre: Stephen.Sarre@canberra.edu.au

Corresponding author:

Elodie Modave, Institute for Applied Ecology, University of Canberra, ACT, 2601, Australia.

Telephone: +61 2 6201 2267. Fax: +61 2 6201 5305. Email: elodie.modave@canberra.edu.au

Running title:

Mini-barcode for species identification

ABSTRACT

Background: Identification of species from trace samples is now possible through the comparison of diagnostic DNA fragments against reference DNA sequence databases. DNA

detection of animals from non-invasive samples, such as predator faeces (scats) that contain traces of DNA from their species of origin, has proved to be a valuable tool for management of elusive wildlife. However, application of this approach can be limited by the availability of appropriate genetic markers. Scat DNA is often degraded, meaning that longer DNA sequences, including standard DNA barcoding markers, are difficult to recover. Instead, targeted short diagnostic markers are required to serve as diagnostic mini-barcodes. The mitochondrial genome is a useful source of such trace DNA markers, because it provides good resolution at species level and occurs in high copy numbers per cell.

Results: We developed a mini-barcode, based on a short (178 bp) fragment of the conserved 12S rRNA mitochondrial gene sequence, with the goal of discriminating amongst the scats of large mammalian predators of Australia. We tested the sensitivity and specificity of our primers and can accurately detect and discriminate amongst quolls, cats, dogs, foxes and devils from trace DNA samples.

Conclusions: Our approach provides a cost effective, time efficient and non-invasive tool that enables identification of all eight medium-large mammal predators in Australia, including native and introduced species, using a single test. With modification, this approach is likely to be of broad applicability elsewhere.

Keywords:

12S rRNA; Dasyurus; DNA barcoding; DNA detection; marsupial; monitoring

BACKGROUND

The looming biodiversity crisis, referred to by some as the Sixth Mass Extinction [1], has made the conservation of wildlife a rapidly growing concern. There is an urgent need to

document the distribution of biodiversity as the foundation for identifying effective solutions to wildlife management issues. The rapid and reliable identification of species at local and regional scales provides the first step towards determining the distribution of biodiversity in the landscape and changes that might be occurring in that distribution.

Advances in genetics and genomics have revolutionized many areas of biology. In particular, the identification of species from trace and environmental samples (e.g. water, soil and faeces, or scats) is now possible through DNA barcoding [2], [3], [4], [5]. The identity of an unknown sample is established by comparisons between DNA sequences obtained from that sample and an appropriate reference sequence database. The application of DNA barcoding for the identification of species from such environmental DNA (eDNA) samples is useful, particularly when the target species is rare, or elusive, where it is difficult to trap or observe without direct interference with live animals, or where morphological identification is problematic [6], [7], [8], [9], [10]. It also makes possible the identification of diet from scats where morphological determinations are likely to be unsuitable for many elements of the diet [11], [12], [13], [14], [15]. Consequently, eDNA analysis from environmental samples collected across a broad spatial and temporal distribution has great potential for enhancing biodiversity management, but is yet to be widely implemented [16], [17].

The DNA associated with environmental samples tends to be of low quantity or quality and can be degraded. To ensure that markers for eDNA detection are specific and sensitive, target sequences, also known as mini-barcodes, should be short (i.e. 100-200 base pairs (bp); [18], [19], [20], [2]) and yet have high discriminatory power [21], [22], [23], [24]. Marker selection therefore needs to account for the range of species likely to be encountered, as well as discriminating among potential sister taxa. Mitochondrial DNA genes

(mtDNA) are usually targeted because they occur in multiple copies in each cell and are therefore more common in trace samples than nuclear sequences, because they can give good resolution of identification at species level, and because their genome is circular, which helps preserving the DNA in some instances. In regions where little is known of the genetic characteristics of the faunal assemblage, identifying the most appropriate DNA sequences to target the fauna present to achieve acceptable levels of accuracy is a challenging exercise and requires a reference database that is sufficiently comprehensive to ensure accurate species assignment [25]. In short, we need DNA barcoding markers that are appropriate to the question being addressed, the ecosystem considered and the taxonomic group studied. Most importantly, if DNA detection is going to be of practical benefit, we need to maximise its effectiveness by developing mini-barcodes that target as many taxa as possible, thus minimising the number of tests that need to be applied. Most barcode tests so far implemented for terrestrial systems have targeted single species (examples in [7], [9], [26], [27]) to avoid the ambiguity that might arise among closely related taxa. Here, we tackle this problem using all extant medium-large Australian mammalian predators as a case study.

Australia has a unique assemblage of medium-large mammalian predators, including a suite of marsupials of Gondwanan heritage intermixed with relatively recently arrived eutherian mammals introduced by humans [28], [29]. Here, we develop a DNA mini-barcode to discriminate amongst these key predators, with the goal of species identification using eDNA extracted from scats. We targeted the top native marsupial predators that are likely to produce large easily visible scats including: six species of quoll (four Australian and two New-Guinean; *Dasyurus maculatus*, *D. viverrinus*, *D. geoffroii*, *D. hallucatus*, *D. albopunctatus* and *D. spartacus*), the Tasmanian devil (*Sarcophilus harrisii*), and the extinct thylacine (*Thylacinus cynocephalus*), as well as key eutherian mammal predators: the native dingo (*Canis lupus*

dingo), and the introduced domestic dog (*Canis lupus familiaris*), red fox, (*Vulpes vulpes),* and domestic cat (*Felis catus*) that are now feral in much of the country. Most of the native marsupial predators have been in decline since, or even before, European settlement in 1788 [30]. Tasmanian devils and the thylacine became extinct on the Australian mainland within the last 3000 to 4000 years [31], but still existed on the island of Tasmania at the time of European settlement. The thylacine has subsequently been hunted to extinction [32], [33] while devil populations have decreased dramatically since the 1990s following the emergence of Devil Facial Tumour Disease [34], [35], [36]. Several species of quoll, together with the dingo, have declined in distribution and abundance on the Australian mainland since European settlement from multiple causes that probably include habitat destruction, hunting, predation by cats and foxes, the spread of cane toads [37], [38], [39] and in the case of dingos, hybridisation with domestic dogs. Although declining or extinct on the mainland, substantial populations of the Tasmanian devil, the spotted-tailed quoll (*D. maculatus)* and the eastern quoll (*D. viverrinus*) remain on the island of Tasmania where they have important ecological roles [40]. However, the recent introduction of foxes to Tasmania [41] and potential competition with feral cats [42], [43] compound the issue, and have stimulated an urgent need to understand threats to native predator populations and enable effective management.

Our goal was to develop a mini-barcode that can identify all medium to large mammal predators in Australia in a single analysis, including identification of all quolls to species level. This has been difficult to achieve using existing genetic markers because of the high levels of sequence conservation observed between quoll species. We compiled a reference tissue collection and identified a mini-barcode based on the conserved 12S rRNA mitochondrial sequence that discriminates among all taxa with the goal of discriminating

among taxa while varying little within species [44], [45]. Two factors generally limit the application of this approach. First, short diagnostic sequences that encompass the range of species to be targeted are difficult to find and are likely to be specific to a particular faunal assemblage. Second, the full suite of potential target organisms tends to be poorly known in most natural systems, and reference DNA sequences are not available for many wildlife species, necessitating the development of reference libraries to guide marker selection and interpretation of results. We evaluate the specificity and sensitivity of this mini-barcode using the framework outlined in [25] and [46]. By targeting all extant medium to large carnivores in Australia, we aim to produce a mini-barcode that can be applied broadly within continental Australia as well as Tasmania. We demonstrate that despite close homology among some taxa, it is possible to design and implement eDNA markers with high discriminatory power for key continental terrestrial fauna incorporating both marsupials and eutherian mammals. Our approach can be implemented in other parts of the world by targeting appropriate fauna assemblage in the development of the mini-barcode.

DATA DESCRIPTION

We identified the 12S rRNA gene as a target for development of a mini-barcode marker. We developed a reference DNA database for this gene, including 174 sequences from 24 genera and 41 mammal species. Sequences were obtained from GenBank, with additional targeted sequencing conducted for species under-represented in GenBank. Sequences were aligned, trimmed to 901 bp, and are provided here in FASTA format (Additional file 1) with additional information on sample and sequence origins in .csv format (Additional file 2).

We used the R package SPIDER [47] to conduct a sliding window analysis to identify a short diagnostic region of the 12S rRNA gene suitable for use as a mini-barcode marker. R code for this analysis is provided in text format (Additional file 3).

Following design of the *AusPreda_12S* primers, we conducted bioinformatic and laboratory evaluations of the sensitivity and specificity of the mini-barcode. We created two modified versions of our reference 12S rRNA database, trimmed to include only the 178 bp flanked by the mini-barcode *AusPreda_12S* primers. The "FULL" database included all 174 sequences from the original database, while the "UNIQUE" database included a subset of 44 sequences, where each haplotype was represented by only a single sequence, and where singleton species (species represented by only one haplotype) were removed. These two databases are provided here in FASTA format (Additional files 4 and 5). We used the R package SPIDER to conduct genetic distance based evaluations of the *AusPreda_12S* primers, to identify the risks of incorrect or ambiguous species identifications based on this sequence. R code for these analyses is provided in text format (Additional file 6) and detailed results are provided in .csv format (Additional file 7).

We conducted PCRs to evaluate amplification success using the *AusPreda_12S* primers on tissue samples from a range of mammal species. Details of samples used are provided in .csv format (Additional file 8). We also tested amplification success from known-origin scats collected from six different predator species. All PCR products successfully amplified from scats were sequenced to confirm predator of origin: resulting sequences are provided here in FASTA format (Additional file 9).

RESULTS

Development of a new mammal mini-barcode

We selected the 12S rRNA gene as a promising candidate marker for development of a mini-barcode and developed a 12S rRNA reference sequence database for Australian mammals comprising 174 sequences. Within the 12S rRNA gene, we identified a 178 bp diagnostic mini-barcode region that displayed high levels of inter-specific variation. We designed conserved primers, *AusPreda_12SF* and *AusPreda_12SR*, to amplify this mini-barcode from a range of mammal species, producing a PCR product of 218 bp in length including primers.

Bioinformatic evaluation of the mini-barcode

We used three different genetic distance based analyses to estimate the risks of species mis-identification when using our *AusPreda_12S* primers on samples of unknown origin (Table 1, Additional file 7). These analyses used versions of the 12S rRNA reference sequence database, trimmed to include only the 178 bp mini-barcode region (Additional files 4 and 5). A *nearNeighbour* analysis of all sequences (the "FULL" database) correctly identified 156 sequences and incorrectly identified 18 sequences. All incorrectly identified sequences originated from species for which only a single reference sequence was available (i.e. singleton species), and thus the nearest neighbour was automatically another species. In most cases this nearest neighbour was a member of the same genus. For example, the nearest neighbour of the only bronze quoll (*D. spartacus*) sequence available was from the western quoll (*D. geoffroii*). This close genetic similarity has also been shown by Woolley *et al.* [48]. Further analyses using a database including only unique haplotypes, from which singleton species were excluded (the "UNIQUE" database) identified correctly all 44 sequences.

Table 1: Summary of results of genetic distance-based evaluations of the *AusPreda_12S* mini-barcode.

	FULL (1% threshold)				UNIQUE (4% threshold)			
	Correct / True	Incorrect / False	Ambiguous	No ID	Correct / True	Incorrect / False	Ambiguous	No ID
Nearest neighbour	155	18	-	-	44	0	-	-
Best close match	147	3	0	24	42	0	0	2
Thresh ID	142	3	5	24	42	0	0	2

Legend: Summary of results of genetic distance-based evaluations of the *AusPreda_12S* mini-barcode conducted using the R package SPIDER to analyse the "FULL" and "UNIQUE" reference sequences databases. The specified genetic distance thresholds were used for the *bestCloseMatch* and *threshID* analyses

BestCloseMatch and ThreshID analyses, which both assume that sequences from a single species fall within a specified genetic distance threshold, correctly identified 147 and 142 sequences respectively in the "FULL" database. Three sequences were incorrectly identified in both analyses: *Dasyurus spartacus* (AF009892), *Pseudantechinus macdonnellensis* (EU086642) and *Pseudantechinus roryi* (EU086650) each representing singleton species, and falling within the 1% genetic distance threshold of a congeneric species enabling them to be mistaken for their close relatives. Five *D. geoffroii* sequences were correctly identified using *BestCloseMatch* but were ambiguously identified in the *ThreshID* analysis because of a close similarity (within the 1% genetic distance threshold) with the single *D. spartacus* sequence. A further 24 sequences could not be identified in either analysis because all other sequences within the reference database were more than 1% different. The majority of these sequences were from singletons, but a more relaxed genetic distance threshold (2%-5%) identified them correctly. *BestCloseMatch* and *ThreshID* analyses of the "UNIQUE" database identified correctly 42 of 44 sequences, but the two remaining sequences, both from *Dasycercus cristicauda*, could not be identified (Table 1; details of results: Additional file 7). As noted previously, these sequences would have been correctly identified if a genetic distance threshold of 5% was used.

Evaluation of the amplification success and sensitivity of the *AusPreda_12S* primers

Our mini-barcode was successfully amplified from all 45 tissue samples tested, including samples from a wide range of Australian mammals, as well as a reptile, an amphibian and a bird (Figure 1, Additional file 8). This demonstrates the broad applicability of the primers across the mammalian taxa.

We also successfully amplified our mini-barcode from a wide range of input template DNA concentrations. We set up serial dilutions of DNA from six predator species. Amplification was successful for all three qPCR replicates from all six species for all dilutions from 9 ng / µl to 9 pg / µl inclusive, demonstrating that the primers can amplify from low quantity DNA. Amplification success was less consistent at the highest and lowest DNA concentrations, estimated at 90 ng / µl, 0.9 pg /µl and 0.09 pg / µl (Table 2) indicating that reliability of predator detection from DNA below 9 pg / µl may be poor. Failure to amplify from highly concentrated DNA, despite successful amplification from dilutions of the same DNA extracts, may reflect the presence of PCR inhibitors in these extracts, which were obtained from museum and roadkill specimens.

Table 2: Results of qPCR tests: determine amplification success of the *AusPreda*_12S mini-barcode from low template DNA.

Species	Dilution	Replicate 1	Replicate 2	Replicate 3	CT Mean[1]
**Cat N22b**	1 in 10 (9 ng/µl)	12.444	14.281	13.373	13.366
	1 in 100 (0.9 ng/µl)	**16.346***	13.399	13.368	13.384
	1 in 1000 (0.09 ng/µl)	19.252	23.382	23.994	22.209
	1 in 10 000 (9 pg/µl)	31.252	27.486	27.604	28.781
	1 in 100 000 (0.9 pg/µl)	31.483	31.476	29.386	30.782
	1 in 1 000 000 (0.09 pg/µl)	**Undetermined**	**Undetermined**	**Undetermined**	-
**Dingo AA15020**	1 in 10 (9 ng/µl)	14.303	13.019	15.363	14.228
	1 in 100 (0.9 ng/µl)	15.879	16.791	16.623	16.431
	1 in 1000 (0.09 ng/µl)	19.719	19.237	17.424	18.793
	1 in 10 000 (9 pg/µl)	22.652	24.957	25.196	24.268
	1 in 100 000 (0.9 pg/µl)	**Undetermined**	**Undetermined**	**Undetermined**	-
	1 in 1 000 000 (0.09 pg/µl)	**Undetermined**	**Undetermined**	**Undetermined**	-
**Eastern quoll UC1214**	1 in 10 (9 ng/µl)	14.128	13.509	13.449	13.695
	1 in 100 (0.9 ng/µl)	17.267	**20.866***	17.235	17.251
	1 in 1000 (0.09 ng/µl)	17.662	21.523	21.385	20.190
	1 in 10 000 (9 pg/µl)	24.346	26.474	25.653	25.491
	1 in 100 000 (0.9 pg/µl)	**Undetermined**	**Undetermined**	34.570	34.570
	1 in 1 000 000 (0.09 pg/µl)	**Undetermined**	**Undetermined**	**Undetermined**	-
**Spotted-tailed quoll A3395**	1 in 10 (9 ng/µl)	13.460	13.928	14.048	13.812
	1 in 100 (0.9 ng/µl)	17.517	16.447	18.653	17.539
	1 in 1000 (0.09 ng/µl)	20.374	19.540	17.003	18.972
	1 in 10 000 (9 pg/µl)	27.511	25.453	23.851	25.605
	1 in 100 000 (0.9 pg/µl)	30.158	30.132	25.107	28.466
	1 in 1 000 000 (0.09 pg/µl)	**Undetermined**	35.172	**Undetermined**	35.172
**Red fox UC0401**	1 in 10 (9 ng/µl)	15.547	15.528	14.628	15.234
	1 in 100 (0.9 ng/µl)	19.566	17.524	16.860	17.983
	1 in 1000 (0.09 ng/µl)	21.915	22.827	22.360	22.367
	1 in 10 000 (9 pg/µl)	26.672	25.460	25.508	25.880
	1 in 100 000 (0.9 pg/µl)	31.672	30.914	28.863	30.483
	1 in 1 000 000 (0.09 pg/µl)	**Undetermined**	31.601	**Undetermined**	31.601
**Tasmanian devil A3357**	1 in 10 (9 ng/µl)	15.502	**16.810***	14.536	15.019
	1 in 100 (0.9 ng/µl)	19.736	18.729	19.702	19.389
	1 in 1000 (0.09 ng/µl)	23.517	22.999	21.591	22.702
	1 in 10 000 (9 pg/µl)	27.216	28.006	24.130	26.451
	1 in 100 000 (0.9 pg/µl)	30.876	30.734	28.977	30.196
	1 in 1 000 000 (0.09 pg/µl)	32.534	**Undetermined**	**Undetermined**	32.534

Legend: Results of qPCR tests conducted to determine amplification success of the *AusPreda*_12S mini-barcode from low template DNA. Numbers represent observed CT values for each replicate qPCR of a series of DNA dilutions.

[1]undetermined results were excluded when calculating mean CT.

*where the qPCR traces were of an irregular shape (three replicates), the replicate was excluded when calculating mean CT.

Evaluation of amplification success from trace samples using known-origin scats

We tested the ability of the *AusPreda_12S* primers to correctly identify the known predator by analysing scats from captive animals. 57 scats were tested and amplified product was obtained from 53 samples. We obtained good quality DNA sequences, ranging from 116 bp to 182 bp in length, from 49 (92%) of these 53 scats (Additional file 9). The species of origin was correctly identified for all 49 samples, with scat DNA sequences matched to appropriate GenBank reference sequences with 97-100% sequence identity (Table 3).

DISCUSSION

Non-invasive environmental DNA-based methods can provide a novel approach to the detection of cryptic animals in large-scale surveys [49], with applications to wildlife management. Such DNA approaches can make important contributions to the ability to detect incursions or monitor established invasive species [50], [51], [41] or to detect very rare or declining species of conservation significance [52][8].

Here, we report a PCR-based mini-barcode test for medium-large Australian mammalian predators. This test can amplify DNA from and discriminate among the four quoll species found in Australia, as well as the Tasmanian devil (the only other extant large marsupial predator) and introduced mammal carnivores with a high level of accuracy. We expect that these primers will also amplify DNA from both species of New Guinean quoll. Previous studies have applied barcoding methods to detect individual species across multiple time points (examples in [53], [54]). Here we have shown that it is also possible to

identify multiple species from a single DNA test, using a straightforward PCR and Sanger sequencing approach. All clear sequences obtained from 49 scats of six target predator species were correctly identified to species level. In the small number of cases where a clear sequence was not obtained from a scat, we found that the sequences obtained were mixed, probably arising from the amplification of two or more species in the same sample. This could arise from cross contamination among samples but is more likely the result of the amplification of prey DNA present in the scat [14], [55]. We have previously observed this phenomenon when using a single species test to detect fox DNA, where rabbit or hare DNA were sometimes erroneously amplified [37]. This demonstrates the need to account for the history of samples analysed (how they were obtained, how fresh they were upon collection, and how samples and DNA extracts were stored) and the importance of a DNA sequencing step in any of these analyses to enable recognition of non-specific PCR amplification. In addition to successful amplification of scat DNA, we demonstrate that our mini-barcode primers can successfully amplify low-template DNA (at least as low as 0.9 pg / µl) from museum samples. This provides further evidence of the utility of this marker for application to eDNA studies.

Whilst DNA metabarcoding may more clearly determine which species are represented by mixed samples, metabarcoding methods are relatively costly and require more specialist equipment, which may not be available to many wildlife managers. In this study, PCR and Sanger sequencing reliably identified the predator of origin for 86% of scat samples, which is likely to be sufficient for many management applications and is a higher success rate than has been reported for several other faecal DNA studies [41], [56]. Using our approach, DNA can be screen for the presence of multiple predator species in a single and inexpensive test, without the need to develop and apply a set of species-specific primers

for each predator of interest. We provide a non-invasive instrument with potential utility for scientists or managers working with endangered or invasive Australian predators, but a similar approach could be used to target predator assemblages in other regions.

The bioinformatic evaluation of our mini-barcode shows that this marker can reliably discriminate among the eight target predator species (eastern, western, northern and spotted-tail quolls, Tasmanian devils, cats, dogs and foxes) in Australia. The close genetic similarity between the bronze quoll (from New Guinea) and the western quoll (from Australia), described above and supported by [48], may pose some problems for reliable species identification from unknown samples, but the different geographic distributions of these two species will likely provide a clear identification in most cases.

Further development of our reference database, to include additional *D. albopunctatus* and *D. spartacus* sequences, will be required to better understand the utility of this test for identification of specimens to species level in New Guinea. Likewise, a better reference database would improve the relevance of this DNA test for application to historic samples. Sequences from the extinct thylacine could be clearly identified in our initial analyses, but this species could not be included in the UNIQUE database for further bioinformatic analysis because only one 12S rRNA haplotype was available. Finally, because we are working with mitochondrial DNA which is maternally inherited, we cannot currently use this test to distinguish between dogs and dingos, in part because of the prevalence of hybrids in many wild populations.

Considerations when working with scats

One important consideration for future studies using the *AusPreda_12S* primers is the need to understand the ecological role of the species from which eDNA is detected.

Typically, predator DNA is the most abundant in scats, owing to the release of epithelial cells during defecation [57], [58], [59]. However, because there are multiple potential sources of DNA in scat samples, it is also possible that these primers will amplify DNA from prey species. In some cases, this will be obvious, for example where the scats of the prey species detected are clearly morphologically different from carnivore scats. However, other results may be more difficult to interpret, for example where mixed sequences, representing two different predator species which could potentially predate upon one another, are obtained from the same sample.

Conservation implications

The *AusPreda_12S* primers provide an opportunity to enhance monitoring of predators across Australia for conservation purposes. For example, western quolls were successfully re-established in Western Australia in 1987 after a recovery plan implemented over 13 years, in areas previously baited with 1080 to remove introduced species [60]. Western quolls from Western Australia were also re-introduced to the Flinders Ranges in South Australia in 2014, and that population is now breeding in the wild, with more than 60 young born since their relocation [61], [62]. Eastern quolls were re-introduced from Tasmania to Mulligans Flat Woodland Sanctuary, in the Australian Capital Territory, in early 2016 [63]. There are also proposals to reintroduce devils to south-eastern mainland Australia to reduce the negative impact that dingo control has on small-mammals through mesopredator release [64], [65], [66], [67]. The development of this mini-barcode now provides a new tool with which to monitor these re-introduced species, and the non-native predators that threaten them, from non-invasive samples.

Future work

In the future, this predator identification tool may be used to model the distribution of predators in Tasmania or mainland Australia, supplementing more traditional data obtained from live trapping and sightings. It is now possible to reliably detect a predator of interest from non-invasive samples. Using the *AusPreda_12S* primers in an initial sample screening step may provide further opportunities to study the diets of each specific predator, by identifying samples to include in targeted metabarcoding studies. This test could also be more broadly useful, with potential application to detection and monitoring of the two New Guinean quoll species.

METHODS

Selection of a candidate marker gene

We compiled initial reference databases for three mitochondrial genes, 12S rRNA, 16S rRNA and ND2, all of which have proven useful for species detection in other studies [59], [68], [69], [70], [71]. These databases used sequences collected mainly from GenBank [72], [73].

We used the R package SPIDER to identify potential mini-barcodes from these initial reference databases. For each gene, we conducted a sliding window analysis with a window size of 20 bp (representing the length of a typical primer) to identify potential sites for marker development. We considered suitable candidate markers to be regions up to 200 bp in length, incorporating two primer sites (each of 20 bp in length) that were well-conserved across all taxa but which flanked a region of 100-200 bp that displayed high levels of inter-specific variation. We were not able to identify any candidate mini-barcode markers that met these criteria from the 16S rRNA and ND2 genes, so all subsequent work was focused on the 12S rRNA gene.

Development of a reference database for the 12S rRNA gene

We constructed a reference database for the 12S rRNA gene. This included representatives of native and introduced Tasmanian mammal predators and their potential prey species, their mainland Australian relatives, livestock and other introduced species (i.e. goat, sheep, horse, wild boar, cow and fallow deer) and humans. Importantly, all six recognised quoll species (four Australian and two New Guinean) were represented (Additional files 1 and 2). The final reference database consisted of 174 sequences representing 41 species from 24 genera. We obtained the majority of sequences from GenBank, but we generated additional sequences from a selection of species that were under-represented in the public database. DNA was extracted from tissue samples from museum specimens, road-killed animals, and western quoll tissues collected during a reintroduction program in the Flinders Ranges (South Australia) involving quolls of Western Australian origin [74]. We used a salting out method [75] with minor modifications as follows. Our lysis buffer included 10% SDS and tissues were digested in a thermomixer for three hours at 56 °C with mixing at 500 rpm. DNA pellets were air dried for 30-60 minutes and re-suspended in 50 µl of ddH_2O. Genomic DNA extracts were quantified using a Nanodrop ND1000 spectrophotometer (Thermo Fischer Scientific) and samples were diluted with ddH2O to a final concentration of *ca* 40 ng/ µl. The entire 12S gene region was amplified by PCR using primers 12C and 12gg (Table 4). PCRs of 25 µl final volume contained 0.4 µM of each primer, 1x MyTaq^{TM} red mix (Bioline) and *ca* 3.2 ng/ µl of genomic DNA. Cycling conditions were: 95 °C for 2 mins; ten cycles of 95 °C for 20 s, a touchdown from 60 °C - 50 °C for 20 s, and 72 °C for 1 min; then 35 cycles of 95 °C for 20 s, 50 °C for 20 s, and 72 °C for 1 min; followed by a final extension at 72 °C for 4 mins. PCR products were visualised on a 1.7% TBE agarose gel (Agarose I: Amresco, Solon, OH, USA) run for 40 mins at 90 V.

Hyperladder 50 bp (Bioline, Australia) was included to serve as a size reference. Amplicons were cleaned using Diffinity rapid tips (Scientific Specialties, Inc., California, USA) and prepared for sequencing following protocols recommended by the Biomolecular Resource Facility (Australian National University) before being sequenced in both directions on a 96 capillary 3730 DNA Analyzer (Applied Biosystems). Forward and reverse sequences for each sample were manually checked, trimmed of primer sequences and low quality bases at the 3' ends, and aligned using Geneious 8.1.7 (Biomatters, Auckland, New Zealand) [76]. The final alignment was 901 bp in length.

Table 4: PCR primers used in this study.

Marker	Sequence (5' – 3')	Amplicon length	Reference
12C & 12GG	12C: AAAGCAAARCACTGAAAATG<br>12GG: TRGGTGTARGCTRRRTGCTTT	1061 bp	[77]
AusPreda_12S	AusPreda_12SF: CCAGCCACCGCGGTCATACG<br>AusPreda_12SR: GCATAGTGGGGTCTCTAATC	218 bp	This study

Development of primers for the mini-barcode

We conducted a sliding window analysis of our 12S rRNA reference database, using the R package SPIDER [47]. This identified a single candidate mini-barcode region within the 12S gene that included a region of less than 200 bp with high levels of inter-specific variation, flanked by highly conserved regions ideal for primer design (Figure 2 and Additional file 3). We designed two primers within the flanking regions: *AusPreda_12SF* (5'-CCAGCCACCGCGGTCATACG-3') and *AusPreda_12SR* (5'-GCATAGTGGGGTCTCTAATC-3') (Table 4). These amplify a product of 218 bp in length (178 bp excluding primers).

Bioinformatic evaluation of the mini-barcode

We used additional functions of the R package SPIDER to estimate the risks of species mis-identification when using our *AusPreda_12S* primers on samples of unknown origin. These analyses were conducted using two versions of our 12S reference database, trimmed to include only the 178 bp of sequences flanked by the *AusPreda_12S* primers. The "FULL" database included all 174 sequences present in the original database (Additional file 4). The "UNIQUE" database was a subset of the "FULL" database in which each haplotype was represented by only a single sequence, and in which singleton species (species represented by only one haplotype) were removed. This included 44 sequences representing 16 species from 12 genera (Additional file 5).

Pairwise genetic distance was calculated for each pair of sequences using the "raw" model. We conducted bioinformatic analyses using the *nearNeighbour*, *bestCloseMatch*, and *threshID* functions to identify the taxa most likely to be misidentified or ambiguously identified using our primers. R code for these analyses is provided in Additional file 6. The *nearNeighbour* function determines, for each sequence in the reference database, whether the most closely related sequence originates from a conspecific, with two outcomes possible: "true" or "false". A genetic distance threshold must be specified for the *bestCloseMatch* and *threshID* functions to account for intra-specific variation. We estimated the most appropriate genetic thresholds to use for the "UNIQUE" and "FULL" databases to be 4% and 1% respectively based on the thresholds with the lowest cumulative error. The *bestCloseMatch* analysis identified the most closely related sequence, within the specified genetic distance threshold, and its species of origin, for each query sequence. The *threshID* analysis extended this, to consider species of origin for all sequences within the genetic distance threshold. These analyses had four possible outcomes: "correct", "incorrect", "ambiguous" and "no identification" [47].

Evaluation of the amplification success and sensitivity of the *AusPreda_12S* primers

We screened a panel of DNA samples from 45 specimens representing 40 species (Additional file 8) to evaluate amplification success of the *AusPreda_12S* primers. DNA was extracted from tissue samples as described above, and amplified with the *AusPreda_12S* primers using the same cycling conditions as for the 12C and 12gg primers above, with PCR products visualised on a 1.7% TBE agarose gel to determine amplification success (Figure 1).

To test the sensitivity of our primers to detect low template DNA samples, we set up serial dilutions of six DNA extracts originating from museum samples, representing each of the six mammal predators that might be detected in Tasmania (Tasmanian devil, eastern quoll, spotted tail quoll, cat, dog and fox). The DNA concentration of each original DNA extraction was determined using a QuBit Fluorometer and the Qubit dsDNA BR Assay Kit (Thermo Fisher) and diluted with ddH_2O if necessary to obtain a starting concentration of 90 ng / µl. We then set up a series of six 10 X dilutions from each of these "undiluted" (90 ng / µl) samples. For each dilution of each sample, we performed three qPCR replicates, each with a total volume of 25 µl including 1X Gold buffer (Applied Biosystems), 2 mM $MgCl^2$, 0.4 mg / ml BSA, 0.4 µM of each primer, 0.6 µl SYBR green (1:2000 Life Technologies nucleic acid gel stain), 0.25 mM of each dNTP, 1 unit of AmpliTaq Gold™ (Applied Biosystems) and 2 µl of the appropriate DNA dilution. qPCRs were conducted using a Viia7 Real-Time PCR system (Thermo Fisher Scientific) with an initial step of 95 °C for 5 mins; followed by 40 cycles of 95 °C for 30 s, 57 °C for 30 s and 72 ° for 30 s. We conducted a comparative CT analysis using the ViiA7 software v1.2.4, with a threshold of 5,000 ΔRn. For each dilution of each DNA sample we calculated the mean CT value and the standard deviation across PCR replicates2.

Evaluation of amplification success from trace samples using known-origin scats

We used previously-extracted DNA from 57 scats of known-origin collected in 2010-2011 from captive animals, including eastern quolls, spotted-tailed quolls, Tasmanian devils, foxes, cats and dogs. DNA was extracted using a combined chelex (Bio Rad Laboratories, Hercules, California, USA) and spin column (Mega quick-spin Total Fragment DNA Purification Kit, Intron Biotechnology) methods [78]. We evaluated amplification success from these samples using the *AusPreda_12S* primers, by conducting PCRs and visualising PCR products by gel electrophoresis as described above.

All amplified products were sequenced in both directions using the *AusPreda_12S* primers, following the methods described above for primers 12C and 12gg. Forward and reverse reads were aligned in Geneious 8.1.7 using a global alignment with free end gaps (Geneious alignment) allowing 65% similarity. Primers were trimmed and a consensus sequence was generated for each sample. Consensus sequences were compared against the GenBank database using nucleotide BLAST (Basic Local Alignment Search Tool, MEGABLAST with the “nr” option and a maximum hit of 20) to identify the most likely species of origin.

Availability of supporting data and material

The datasets and R code associated with this article are provided as supporting information. All DNA sequences generated during this study have been submitted to GenBank: accession numbers KX786294 to KX786344.

Additional file 1: 12S rRNA reference sequence database used for primer design (FASTA format)

Additional file 2: Samples included in the 12S rRNA reference sequence database used for primer design (.csv format)

Additional file 3: R code for sliding windows analysis implemented using SPIDER (text format)

Additional file 4: Reference database used for genetic distance based evaluation of the *AusPreda_12S* mini-barcode: “FULL” database (FASTA format)

Additional file 5: Reference database used for genetic distance based evaluation of the *AusPreda_12S* mini-barcode: “UNIQUE” database (FASTA format)

Additional file 6: R code for genetic distance based evaluation of the *AusPreda_12S* mini-barcode implemented using SPIDER (text format)

Additional file 7: Detailed results of genetic distance based evaluation of the *AusPreda_12S* mini-barcode (.csv format)

Additional file 8: Samples included in the laboratory evaluation of the *AusPreda_12S* mini-barcode (.csv format)

Additional file 9: Consensus sequences obtained from 53 known-origin scats by amplification with the *AusPreda_12S* mini-barcode (FASTA format)

The datasets supporting results of this article are available in the X repository, #

List of abbreviations

BLAST: Basic Local Alignment Search Tool: Tool available through NCBI to compare an unknown sequence to existing sequences in a public database.

bp: base pairs: pairs of nucleotides in a DNA or RNA strand

CT value: cycle threshold: the number of cycles required for the fluorescent signal of a qPCR machine to cross the predetermined threshold.

DNA: deoxyribonucleic acid

mtDNA: mitochondrial DNA

eDNA: environmental DNA

PCR: polymerase chain reaction, a method used to amplify a target DNA or RNA strand

rRNA: ribosomal ribonucleic acid

TBE: Tris/Borate/EDTA: buffer for gel electrophoresis

Consent for publication

Not applicable

Competing interests

Not applicable

Funding

This study was funded by the Invasive Animals Cooperative Research Centre as part of project 1.L.21.

Authors' contributions

EM, AM and SS designed the study. EM performed the experiments. EM and AM analysed the data. EM wrote the manuscript and AM and SS provided extensive comments. All authors read and approved the final manuscript.

Acknowledgements

Thanks to the following for providing samples: Alison Taylor (Kippax Veterinary Hospital); Kejun Wei (Australian National University); Sally Bryant, Elise Dewar and Michael Driessen (Department of Primary Industries, Parks, Water and Environment, Tasmania); Shane McHugh (Tasmanian Land Conservancy); Judy Dunlop (Department of Parks and Wildlife, Western Australia); Leo Joseph and Robert Palmer (Australian National Wildlife Collection,

CSIRO); Melissa Jensen (University of Adelaide); Catriona Campbell and Matthew Young (University of Canberra); Tony Buckmaster (University of Sydney); Shane Jackson (University of Tasmania); Phil Newman (Rugosa Park Alpacas); Conrad Barr; The Australian Museum; Museum Victoria; The Tasmanian Museum and Art Gallery; The Queen Victoria Museum and Art Gallery. Special thanks to Tony Buckmaster, Arthur Georges and members of the Invasive Animals CRC's 2016 Kioloa writing workshop as well as Stephen Harris for helpful suggestions.

REFERENCES

[1] G. Ceballos, P. R. Ehrlich, A. D. Barnosky, A. García, R. M. Pringle, and T. M. Palmer, "Accelerated modern human–induced species losses: Entering the sixth mass extinction," *Sci. Adv.*, vol. 1, no. 5, p. e1400253, 2015.

[2] S. Boyer, S. D. J. Brown, R. A. Collins, R. H. Cruickshank, M.-C. Lefort, J. Malumbres-Olarte, S. D. Wratten, and K. A. Crandall, "Sliding window analyses for optimal selection of mini-barcodes, and application to 454-pyrosequencing for specimen identification from degraded DNA," *PLoS One*, vol. 7, no. 5, 2012.

[3] I. Meusnier, G. A. C. Singer, J.-F. Landry, D. A. Hickey, P. D. N. Hebert, and M. Hajibabaei, "A universal DNA mini-barcode for biodiversity analysis," *BMC Genomics*, vol. 9, no. 1, p. 214, 2008.

[4] A. Alberdi, I. Garin, O. Aizpurua, and J. Aihartza, "The foraging ecology of the mountain long-eared bat Plecotus macrobullaris revealed with DNA mini-barcodes," *PLoS One*, vol. 7, no. 4, p. e35692, 2012.

[5] W. J. Kress, C. García-Robledo, M. Uriarte, and D. L. Erickson, "DNA barcodes for ecology, evolution, and conservation," *Trends Ecol. Evol.*, no. 0, 2015.

[6] O. Berry and S. D. Sarre, "Gel-free species identification using melt-curve analysis," *Mol. Ecol. Notes*, vol. 7, no. 1, pp. 1–4, 2007.

[7] T. M. Wilcox, K. S. McKelvey, M. K. Young, S. F. Jane, W. H. Lowe, A. R. Whiteley, and M. K. Schwartz, "Robust detection of rare species using environmental DNA: the importance of primer specificity," *PLoS One*, vol. 8, no. 3, p. e59520, 2013.

[8] H. C. Rees, B. C. Maddison, D. J. Middleditch, J. R. M. Patmore, and K. C. Gough, "REVIEW: The detection of aquatic animal species using environmental DNA–a review of eDNA as a survey tool in ecology," *J. Appl. Ecol.*, vol. 51, no. 5, pp. 1450–1459, 2014.

[9] O. Berry, S. D. Sarre, L. Farrington, and N. Aitken, "Faecal DNA detection of invasive species: The case of feral foxes in Tasmania," *Wildl. Res.*, vol. 34, no. 1, pp. 1–7, 2007.

[10] J. M. Korstian, A. M. Hale, V. J. Bennett, and D. A. Williams, “Using DNA barcoding to improve bat carcass identification at wind farms in the United States,” *Conserv. Genet. Resour.*, pp. 1–8, 2015.

[11] W. Shehzad, T. M. McCarthy, F. Pompanon, L. Purevjav, E. Coissac, T. Riaz, and P. Taberlet, “Prey preference of snow leopard (*Panthera uncia*) in South Gobi, Mongolia,” *PLoS One*, vol. 7, no. 2, p. e32104, 2012.

[12] M. Koester, S. Claßen, and R. Gergs, “Establishment of group-specific PCR primers for the identification of freshwater macroinvertebrates,” *Conserv. Genet. Resour.*, vol. 5, no. 4, pp. 1091–1093, 2013.

[13] S. K. Gupta and A. Kumar, “Molecular identification of man-eating carnivores from scat samples,” *Conserv. Genet. Resour.*, vol. 6, no. 2, pp. 271–274, 2014.

[14] F. Grattarola, S. González, and M. Cosse, “A novel primer set for mammal species identification from feces samples,” *Conserv. Genet. Resour.*, vol. 7, no. 1, pp. 57–59, 2015.

[15] C. Shores, S. Mondol, and S. K. Wasser, “Comparison of DNA and hair-based approaches to dietary analysis of free-ranging wolves (Canis lupus),” *Conserv. Genet. Resour.*, vol. 7, no. 4, pp. 871–878, 2015.

[16] T. Takahara, T. Minamoto, and H. Doi, “Using environmental DNA to estimate the distribution of an invasive fish species in ponds,” *PLoS One*, vol. 8, no. 2, p. e56584, 2013.

[17] A. Fujiwara, S. Matsuhashi, H. Doi, S. Yamamoto, and T. Minamoto, “Use of environmental DNA to survey the distribution of an invasive submerged plant in ponds,” *Freshw. Sci.*, vol. 35, no. 2, p. 0, 2016.

[18] B. E. Deagle, J. P. Eveson, and S. N. Jarman, “Quantification of damage in DNA recovered from highly degraded samples - A case study on DNA in faeces,” *Front. Zool.*, vol. 3, 2006.

[19] M. Hajibabaei, M. Smith, D. H. Janzen, J. J. Rodriguez, J. B. Whitfield, and P. D. N. Hebert, “A minimalist barcode can identify a specimen whose DNA is degraded,” *Mol. Ecol. Notes*, vol. 6, no. 4, pp. 959–964, 2006.

[20] A. Valentini, F. Pompanon, and P. Taberlet, “DNA barcoding for ecologists,” *Trends Ecol. Evol.*, vol. 24, no. 2, pp. 110–117, 2009.

[21] A. R. Bahrmand, H. Madani, V. V Bakayev, M. H. Babaei, G. Samar, and V. Anashchenko, “Polymerase chain reaction of bacterial genomes with single universal primer: application to distinguishing mycobacteria species,” *Mol. Cell. Probes*, vol. 10, no. 2, pp. 117–122, 1996.

[22] R. Meier, K. Shiyang, G. Vaidya, and P. K. L. Ng, “DNA barcoding and taxonomy in Diptera: a tale of high intraspecific variability and low identification success,” *Syst. Biol.*, vol. 55, no. 5, pp. 715–728, 2006.

[23] R. Boutros, N. Stokes, M. Bekaert, and E. C. Teeling, “UniPrime2: a web service providing easier Universal Primer design,” *Nucleic Acids Res.*, p. gkp269, 2009.

[24] E. M. Furlan, D. Gleeson, C. M. Hardy, and R. P. Duncan, “A framework for estimating the sensitivity of eDNA surveys,” *Mol. Ecol. Resour.*, 2015.

[25] A. J. Macdonald and S. D. Sarre, “A framework for developing and validating taxon-specific primers for specimen identification from environmental DNA,” *Mol. Ecol. Resour.*, 2016.

[26] R. E. Wheat, J. M. Allen, S. D. L. Miller, C. C. Wilmers, and T. Levi, “Environmental DNA from residual saliva for efficient noninvasive genetic monitoring of brown bears (Ursus arctos),” *PLoS One*, vol. 11, no. 11, p. e0165259, 2016.

[27] D. J. Morin, M. J. Kelly, and L. P. Waits, “Monitoring coyote population dynamics with fecal DNA and spatial capture–recapture,” *J. Wildl. Manage.*, vol. 80, no. 5, pp. 824–836, 2016.

[28] J. C. Z. Woinarski, A. A. Burbidge, and P. L. Harrison, “Ongoing unraveling of a continental fauna: decline and extinction of Australian mammals since European settlement,” *Proc. Natl. Acad. Sci.*, vol. 112, no. 15, pp. 4531–4540, 2015.

[29] A. A. Burbidge and N. L. McKenzie, “Patterns in the modern decline of Western Australia’s vertebrate fauna: causes and conservation implications,” *Biol. Conserv.*, vol. 50, no. 1–4, pp. 143–198, 1989.

[30] A. P. Elkin, “Reaction and interaction: a food gathering people and European settlement in Australia,” *Am. Anthropol.*, vol. 53, no. 2, pp. 164–186, 1951.

[31] O. J. F. Brown, “Tasmanian devil (Sarcophilus harrisii) extinction on the Australian mainland in the mid-Holocene: multicausality and ENSO intensification,” *Alcheringa An Australas. J. Palaeontol.*, vol. 30, no. S1, pp. 49–57, 2006.

[32] F. W. King, “Extant unless proven extinct: the international legal precedent,” *Conserv. Biol.*, vol. 2, no. 4, pp. 395–397, 1988.

[33] R. Paddle, *The last Tasmanian tiger: the history and extinction of the thylacine*. Cambridge University Press, 2002.

[34] T. Hollings, M. Jones, N. Mooney, and H. McCallum, “Trophic Cascades Following the Disease-Induced Decline of an Apex Predator, the Tasmanian Devil,” *Conserv. Biol.*, 2013.

[35] A. Brüniche-Olsen, C. P. Burridge, J. J. Austin, and M. E. Jones, “Disease induced changes in gene flow patterns among Tasmanian devil populations,” *Biol. Conserv.*, vol. 165, pp. 69–78, 2013.

[36] H. S. Bender, J. A. Marshall Graves, and J. E. Deakin, “Pathogenesis and molecular biology of a transmissible tumor in the Tasmanian devil,” *Annu. Rev. Anim. Biosci.*, vol. 2, no. 1, pp. 165–187, 2014.

[37] S. Burnett, “Colonizing cane toads cause population declines in native predators: reliable anecdotal information and management implications,” *Pacific Conserv. Biol.*, vol. 3, no. 1, p. 65, 1997.

[38] C. A. Belcher, “Demographics of tiger quoll (*Dasyurus maculatus maculatus*) populations in south-eastern Australia,” *Aust. J. Zool.*, vol. 51, no. 6, pp. 611–626, Jan. 2003.

[39] A. S. Glen, P. J. de Tores, D. R. Sutherland, and K. D. Morris, “Interactions between chuditch (Dasyurus geoffroii) and introduced predators: a review,” *Australian Journal of Zoology*, vol. 57 , no. 5. pp. 347–356, 2009.

[40] B. A. Fancourt, C. E. Hawkins, and S. C. Nicol, “Evidence of rapid population decline of the eastern quoll (Dasyurus viverrinus) in Tasmania,” *Aust. Mammal.*, vol. 35, no. 2, pp. 195–205, 2013.

[41] S. D. Sarre, A. J. Macdonald, C. Barclay, G. R. Saunders, and D. S. L. Ramsey, “Foxes are now widespread in Tasmania: DNA detection defines the distribution of this rare but invasive carnivore,” *J. Appl. Ecol.*, vol. 50, no. 2, pp. 459–468, 2013.

[42] M. Nogales, A. Martín, B. R. Tershy, C. J. Donlan, D. Veitch, N. Puerta, B. Wood, and J. Alonso, “A review of feral cat eradication on islands,” *Conserv. Biol.*, vol. 18, no. 2, pp. 310–319, 2004.

[43] B. T. Lazenby, N. J. Mooney, and C. R. Dickman, “Effects of low-level culling of feral cats in open populations: a case study from the forests of southern Tasmania,” *Wildl. Res.*, vol. 41, no. 5, pp. 407–420, 2015.

[44] A. R. Palumbi and F. Cipriano, “Species identification using genetic tools: the value of nuclear and mitochondrial gene sequences in whale conservation,” *J. Hered.*, vol. 89, no. 5, pp. 459–464, 1998.

[45] D. M. Hillis, C. Moritz, B. K. Mable, and R. G. Olmstead, *Molecular systematics*, vol. 23. Sinauer Associates Sunderland, MA, 1996.

[46] A. J. MacDonald and S. D. Sarre, “Species assignment from trace DNA sequences: an in silico assessment of the test used to survey for foxes in Tasmania,” *J. Appl. Ecol.*, p. n/a–n/a, Jul. 2015.

[47] S. D. J. Brown, R. A. Collins, S. Boyer, M. LEFORT, J. MALUMBRES-OLARTE, C. J. Vink, and R. H. Cruickshank, “Spider: an R package for the analysis of species identity and evolution, with particular reference to DNA barcoding,” *Mol. Ecol. Resour.*, vol. 12, no. 3, pp. 562–565, 2012.

[48] P. A. Woolley, C. Krajewski, and M. Westerman, “Phylogenetic relationships within Dasyurus (Dasyuromorphia: Dasyuridae): quoll systematics based on molecular evidence and male characteristics,” *J. Mammal.*, vol. 96, no. 1, pp. 37–46, Mar. 2015.

[49] M. K. Schwartz, G. Luikart, and R. S. Waples, “Genetic monitoring as a promising tool for conservation and management,” *Trends Ecol. Evol.*, vol. 22, no. 1, pp. 25–33, 2007.

[50] J. A. Darling and M. J. Blum, “DNA-based methods for monitoring invasive species: a review and prospectus,” *Biol. Invasions*, vol. 9, no. 7, pp. 751–765, 2007.

[51] A. D. Bastos, D. Nair, P. J. Taylor, H. Brettschneider, F. Kirsten, E. Mostert, E. Von Maltitz, J. M. Lamb, P. Van Hooft, and S. R. Belmain, “Genetic monitoring detects an overlooked cryptic species and reveals the diversity and distribution of three invasive Rattus congeners in South Africa,” *BMC Genet.*, vol. 12, no. 1, p. 1, 2011.

[52] C. L. Jerde, A. R. Mahon, W. L. Chadderton, and D. M. Lodge, “‘Sight-unseen’ detection of rare aquatic species using environmental DNA,” *Conserv. Lett.*, vol. 4, no. 2, pp. 150–157, 2011.

[53] N. Fernández, M. Delibes, and F. Palomares, “Landscape evaluation in conservation: molecular sampling and habitat modeling for the Iberian lynx,” *Ecol. Appl.*, vol. 16, no. 3, pp. 1037–1049, 2006.

[54] K. S. McKelvey, J. V. O. N. KIENAST, K. B. Aubry, G. M. Koehler, B. T. Maletzke, J. R. Squires, E. L. Lindquist, S. Loch, and M. K. Schwartz, “DNA analysis of hair and scat collected along snow tracks to document the presence of Canada lynx,” *Wildl. Soc. Bull.*, vol. 34, no. 2, pp. 451–455, 2006.

[55] R. Hausknecht, R. Gula, B. Pirga, and R. Kuehn, “Urine—a source for noninvasive genetic monitoring in wildlife,” *Mol. Ecol. Notes*, vol. 7, no. 2, pp. 208–212, 2007.

[56] Y.-C. Cheng and C.-P. Lin, “Dietary Niche Partitioning of Euphaea formosa and Matrona cyanoptera (Odonata: Zygoptera) on the Basis of DNA Barcoding of Larval Feces,” *J. Insect Sci.*, vol. 16, no. 1, p. 73, 2016.

[57] W. O. C. Symondson, “Molecular identification of prey in predator diets,” *Mol. Ecol.*, vol. 11, no. 4, pp. 627–641, 2002.

[58] S. N. Jarman, B. E. Deagle, and N. J. Gales, “Group-specific polymerase chain reaction for DNA-based analysis of species diversity and identity in dietary samples.,” *Mol. Ecol.*, vol. 13, no. 5, pp. 1313–22, May 2004.

[59] B. E. Deagle, D. J. Tollit, S. N. Jarman, M. A. Hindell, A. W. Trites, and N. J. Gales, “Molecular scatology as a tool to study diet: analysis of prey DNA in scats from captive Steller sea lions,” *Mol. Ecol.*, vol. 14, no. 6, pp. 1831–1842, 2005.

[60] K. Morris, B. Johnson, P. Orell, G. Gaikhorst, A. Wayne, and D. Moro, “Recovery of the threatened chuditch (Dasyurus geoffroii): a case study,” *Predators with Pouches Biol. Carniv. Marsupials. CSIRO Publ. Melb.*, pp. 435–451, 2003.

[61] Department of the Environment, “Western quolls – Reintroducing the species to the Flinders Ranges (SA),” 2015. [Online]. Available: http://www.environment.gov.au/biodiversity/threatened/publications/factsheet-western-quolls.

[62] S. Katsineris, “Endangered Quolls re-introduced to the flinders ranges,” 2015.

[63] E. Hunt, “Eastern quolls return to Australian mainland after more than 50 years,” *The Guardian*, 02-Mar-2016.

[64] A. S. Glen and C. R. Dickman, “Complex interactions among mammalian carnivores in Australia, and their implications for wildlife management,” *Biol. Rev. Camb. Philos. Soc.*, vol. 80, no. 3, pp. 387–401, 2005.

[65] C. N. Johnson and J. VanDerWal, “Evidence that dingoes limit abundance of a mesopredator in eastern Australian forests,” *J. Appl. Ecol.*, vol. 46, no. 3, pp. 641–646, 2009.

[66] E. G. Ritchie and C. N. Johnson, “Predator interactions, mesopredator release and biodiversity conservation,” *Ecol. Lett.*, vol. 12, pp. 982–998, 2009.

[67] D. O. Hunter, T. Britz, M. Jones, and M. Letnic, “Reintroduction of Tasmanian devils to mainland Australia can restore top-down control in ecosystems where dingoes have been extirpated,” *Biol. Conserv.*, vol. 191, pp. 428–435, 2015.

[68] A. Di Finizio, G. Guerriero, G. L. Russo, and G. Ciarcia, “Identification of gadoid species (Pisces, Gadidae) by sequencing and PCR–RFLP analysis of mitochondrial 12S and 16S rRNA gene fragments,” *Eur. Food Res. Technol.*, vol. 225, no. 3–4, pp. 337–344, 2007.

[69] F. Pompanon, B. E. Deagle, W. O. C. Symondson, D. S. Brown, S. N. Jarman, and P. Taberlet, “Who is eating what: diet assessment using next generation sequencing.,” *Mol. Ecol.*, vol. 21, no. 8, pp. 1931–50, Apr. 2012.

[70] P. Taberlet, E. Coissac, F. Pompanon, C. Brochmann, and E. Willerslev, “Towards next-generation biodiversity assessment using DNA metabarcoding,” *Mol. Ecol.*, vol. 21, no. 8, pp. 2045–2050, 2012.

[71] B. E. Deagle, S. N. Jarman, E. Coissac, F. Pompanon, and P. Taberlet, “DNA metabarcoding and the cytochrome c oxidase subunit I marker: not a perfect match,” *Biol. Lett.*, vol. 10, no. 9, p. 20140562, 2014.

[72] “NCBI - National Center for Biotechnology Information.” [Online]. Available: http://www.ncbi.nlm.nih.gov/genbank/. [Accessed: 19-Jan-2017].

[73] L. Y. Geer, A. Marchler-Bauer, R. C. Geer, L. Han, J. He, S. He, C. Liu, W. Shi, and S. H. Bryant, “The NCBI biosystems database,” *Nucleic Acids Res.*, p. gkp858, 2009.

[74] K. E. Moseby, D. E. Peacock, and J. L. Read, “Catastrophic cat predation: A call for predator profiling in wildlife protection programs,” *Biol. Conserv.*, vol. 191, pp. 331–340, 2015.

[75] A. J. MacDonald, S. D. Sarre, N. N. FitzSimmons, and N. Aitken, “Determining microsatellite genotyping reliability and mutation detection ability: an approach using small-pool PCR from sperm DNA,” *Mol. Genet. Genomics*, vol. 285, no. 1, pp. 1–18, 2011.

[76] M. Kearse, R. Moir, A. Wilson, S. Stones-Havas, M. Cheung, S. Sturrock, S. Buxton, A. Cooper, S. Markowitz, and C. Duran, “Geneious Basic: an integrated and extendable desktop software platform for the organization and analysis of sequence data,” *Bioinformatics*, vol. 28, no. 12, pp. 1647–1649, 2012.

[77] M. S. Springer, L. J. Hollar, and A. Burk, “Compensatory substitutions and the evolution of the mitochondrial 12S rRNA gene in mammals.,” *Mol. Biol. Evol.* , vol. 12 , no. 6 , pp. 1138–1150, Nov. 1995.

[78] D. S. L. Ramsey, A. J. MacDonald, S. Quasim, C. Barclay, and S. D. Sarre, “An examination of the accuracy of a sequential PCR and sequencing test used to detect the incursion of an invasive species: the case of the red fox in Tasmania,” *J. Appl. Ecol.*, vol. 52, no. 3, pp. 562–570, 2015.

TABLE

Table 3: PCR and DNA sequencing results from 57 known-origin scat samples screened using the *AusPreda_12S* mini-barcode.

Sample	Scientific name	Common name	Amplified	Sequenced	Closest sequence match using BLAST	% ID$^{\alpha}$	e value$^{\beta}$
100111-27	*Canis lupus familiaris*	Dog	Y	Y	Dog	99.4	1.55E-84
120111-02	*Canis lupus familiaris*	Dog	Y	Y	Dog	100	6.52E-78
121010-11	*Canis lupus familiaris*	Dog	Y	Y	Dog	99.4	1.22E-85
121010-16	*Canis lupus familiaris*	Dog	Y	Y	Dog	98.4	2.08E-83
121010-17	*Canis lupus familiaris*	Dog	Y	Y	Dog	99.4	1.98E-83
121010-30	*Canis lupus familiaris*	Dog	Y	Y	Dog	99.4	5.54E-84
121010-52	*Canis lupus familiaris*	Dog	Y	**N**	**NA**	**NA**	**NA**
121010-53	*Canis lupus familiaris*	Dog	Y	Y	Dog	98.9	2.60E-82
121010-54	*Canis lupus familiaris*	Dog	Y	Y	Dog	99.4	1.22E-85
121010-56	*Canis lupus familiaris*	Dog	Y	Y	Dog	98.9	7.22E-83
121110-55	*Canis lupus familiaris*	Dog	Y	Y	Dog	99.4	5.54E-84
170211-12	*Canis lupus familiaris*	Dog	**N**	**NA**	**NA**	**NA**	**NA**
041110-66	*Dasyurus maculatus*	Spotted-tailed quoll	Y	Y	Spotted-tailed quoll	98.4	2.08E-83
101110-9	*Dasyurus maculatus*	Spotted-tailed quoll	Y	Y	Spotted-tailed quoll	98.2	2.33E-72
170211-25	*Dasyurus maculatus*	Spotted-tailed quoll	Y	Y	Spotted-tailed quoll	99.4	1.55E-84
041110-01	*Dasyurus viverrinus*	Eastern quoll	Y	Y	Eastern quoll	99.4	2.25E-72
041110-04	*Dasyurus viverrinus*	Eastern quoll	Y	Y	Eastern quoll	100	2.05E-88
041110-07	*Dasyurus viverrinus*	Eastern quoll	Y	Y	Eastern quoll	100	4.80E-74
041110-15	*Dasyurus viverrinus*	Eastern quoll	Y	Y	Eastern quoll	100	1.01E-54
041110-74	*Dasyurus viverrinus*	Eastern quoll	Y	Y	Eastern quoll	100	1.19E-85
041110-80	*Dasyurus viverrinus*	Eastern quoll	Y	Y	Eastern quoll	100	9.34E-87
100111-05	*Dasyurus viverrinus*	Eastern quoll	Y	Y	Eastern quoll	100	3.34E-86
100111-31	*Dasyurus viverrinus*	Eastern quoll	Y	Y	Eastern quoll	100	3.34E-86

120111-32	*Dasyurus viverrinus*	Eastern quoll	**N**	**NA**	**NA**	**NA**	**NA**
120111-33	*Dasyurus viverrinus*	Eastern quoll	Y	Y	Eastern quoll	100	2.61E-87
170211-14	*Dasyurus viverrinus*	Eastern quoll	Y	Y	Eastern quoll	100	2.61E-87
100111-04	*Felis catus*	Feral cat	Y	Y	Feral cat	100	1.54E-79
120111-10	*Felis catus*	Feral cat	Y	Y	Feral cat	100	1.56E-79
120111-12	*Felis catus*	Feral cat	Y	Y	Feral cat	100	1.58E-79
120111-31	*Felis catus*	Feral cat	Y	**N**	**NA**	**NA**	**NA**
170211-13	*Felis catus*	Feral cat	Y	Y	Feral cat	99.2	3.36E-60
170211-21	*Felis catus*	Feral cat	Y	Y	Feral cat	100	1.61E-79
170211-22	*Felis catus*	Feral cat	Y	Y	Feral cat	100	1.55E-79
041110-42	*Sarcophilus harrisii*	Tasmanian devil	Y	Y	Tasmanian devil	100	4.02E-80
041110-47	*Sarcophilus harrisii*	Tasmanian devil	Y	Y	Tasmanian devil	100	9.34E-87
041110-48	*Sarcophilus harrisii*	Tasmanian devil	Y	Y	Tasmanian devil	100	2.61E-87
041110-53	*Sarcophilus harrisii*	Tasmanian devil	Y	Y	Tasmanian devil	100	2.47E-82
041110-59	*Sarcophilus harrisii*	Tasmanian devil	Y	Y	Tasmanian devil	100	7.32E-88
121010-06	*Sarcophilus harrisii*	Tasmanian devil	Y	Y	Tasmanian devil	100	4.02E-80
121010-22	*Sarcophilus harrisii*	Tasmanian devil	Y	Y	Tasmanian devil	99.4	5.58E-84
200910-24	*Sarcophilus harrisii*	Tasmanian devil	Y	Y	Tasmanian devil	100	9.34E-87
200910-25	*Sarcophilus harrisii*	Tasmanian devil	Y	Y	Tasmanian devil	100	2.61E-87
080211-04	*Vulpes vulpes*	Red fox	Y	Y	Red fox	99.4	1.22E-85
080211-05	*Vulpes vulpes*	Red fox	Y	Y	Red fox	99.4	5.54E-84
080211-06	*Vulpes vulpes*	Red fox	Y	**N**	**NA**	**NA**	**NA**
080211-07	*Vulpes vulpes*	Red fox	Y	Y	Red fox	97.2	9.35E-61
080211-08	*Vulpes vulpes*	Red fox	Y	Y	Red fox	99.4	5.54E-84
080211-09	*Vulpes vulpes*	Red fox	**N**	**NA**	**NA**	**NA**	**NA**
080211-10	*Vulpes vulpes*	Red fox	Y	Y	Red fox	100	6.52E-78
080211-11	*Vulpes vulpes*	Red fox	Y	Y	Red fox	98.9	5.66E-84

080211-12	*Vulpes vulpes*	Red fox	Y	**N**	**NA**	**NA**	**NA**
080211-13	*Vulpes vulpes*	Red fox	Y	Y	Red fox	98.8	3.99E-75
080211-14	*Vulpes vulpes*	Red fox	Y	Y	Red fox	100	6.52E-78
080211-15	*Vulpes vulpes*	Red fox	Y	Y	Red fox	99.1	2.63E-50
080211-16	*Vulpes vulpes*	Red fox	Y	Y	Red fox	100	6.52E-78
080211-17	*Vulpes vulpes*	Red fox	**N**	**NA**	**NA**	**NA**	**NA**
080211-18	*Vulpes vulpes*	Red fox	Y	Y	Red fox	97.8	1.23E-80

[α] % ID is the percentage pairwise identity between the query sequence and the matching sequence identified using BLAST.

[β] The e-value represents the number of BLAST hits expected by chance. The lower the e-value is, the better.

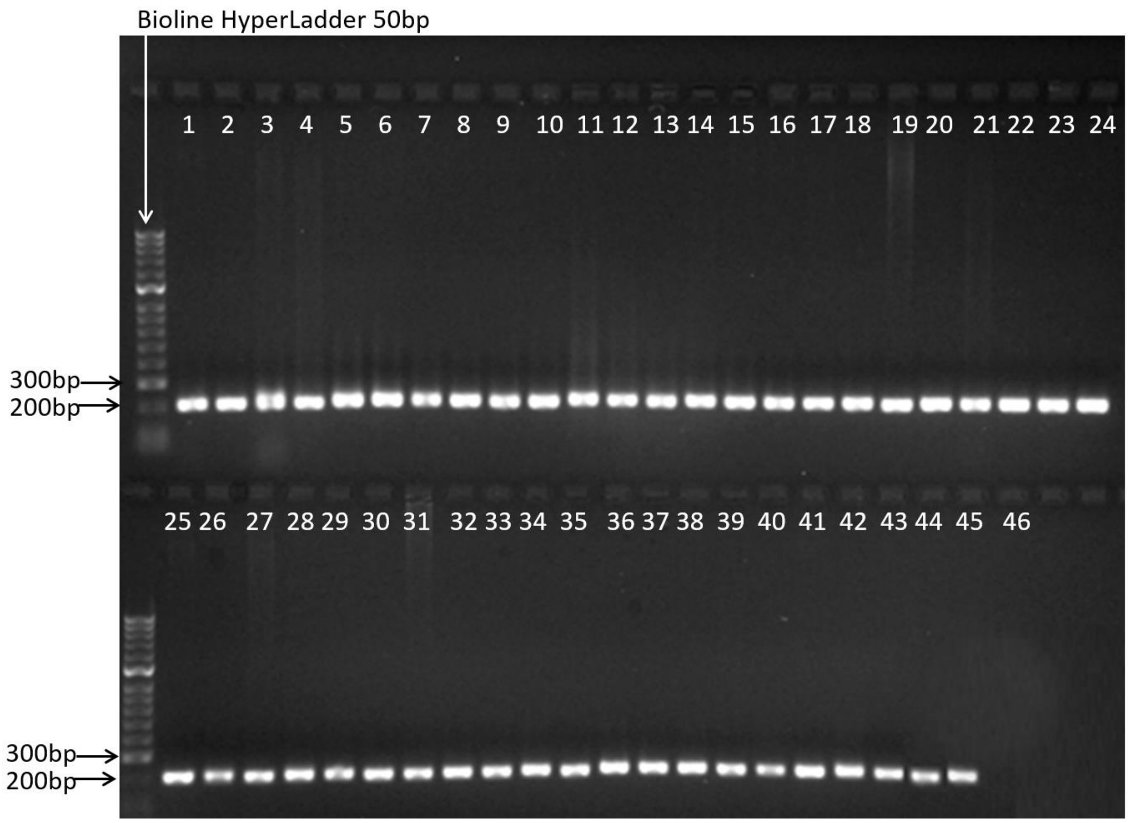

Figure 1: Gel showing amplification success from 45 known tissue samples representing 40 species, using the *AusPreda_12S* mini-barcode primers developed in this study, and a PCR negative. The expected amplicon size is 218bp. Samples are grouped by species as follows: lanes 1 and 2: *Felis catus*, 3: *Canis lupus familiaris*, 4: *Canis lupus dingo*, 5 and 6: *Dasyurus viverrinus*, 7 and 8: *Dasyurus maculatus*, 9 and 10: *Vulpes vulpes*, 11 and 12: *Sarcophilus harrisii*, 13: *Oryctolagus cuniculus*, 14: *Lepus capensis*, 15: *Bos Taurus*, 16: *Ornithorhyncus anatinus*, 17: *Trichosorus vulpecula*, 18: *Petaurus breviceps*, 19: *Tachyglossus aculeatus*, 20: *Potorous tridactylus*, 21: *Bettongia gaimardi*, 22: *Dactylopsila trivirgata*, 23: *Burramys parvus*, 24: *Macropus rufogriseus*, 25: *Thylogale billardierii*, 26: *Pseudomys gracilacaudatus*, 27: *Pseudocheirus peregrinus*, 28: *Antechinus minimus*, 29: *Tiliqua nigrolutea*, 30: *Vombatus ursinus*, 31: *Isoodon obesulus*, 32: *Macropus giganteus*, 33: *Parameles gunnii*, 34: *Sminthopsis leucopus*, 35: *Mus musculus*, 36: *Planigale gilesi*, 37: *Rattus lutreolus velutinus*, 38: *Phascogale tapoatafa*, 39: *Hydromys chrysogaster*, 40: *Macropus rufus*, 41: *Vicugna pacos*, 42: *Dasyurus hallucatus*, 43: *Lathamus discolour*, 44: *Geocrinia laevis*, 45: *Dasyurus geoffroii*, 46: PCR negative.

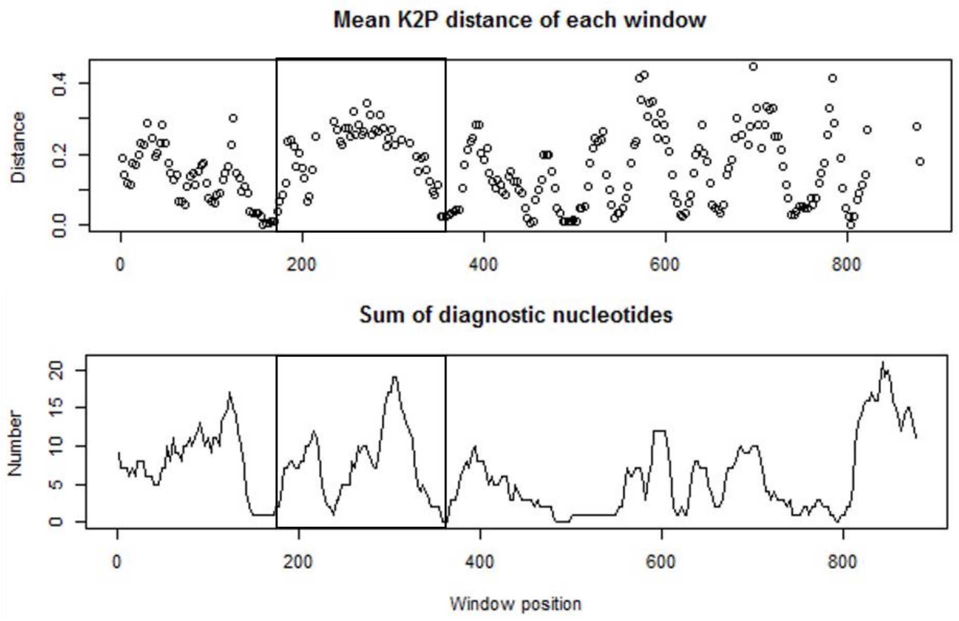

Figure 2: Results of the sliding window analysis conducted using the R package SPIDER for the 12S rRNA gene using a window size of 20 bp. The upper panel displays the mean K2P distance calculated for each 20 bp window. K2P distances are a measure of genetic differentiation among species, with K2P values represented on the y-axis. The lower panel displays the number of diagnostic nucleotides among species identified within each 20 bp window. In both cases, the x-axis represents the position of each window in the alignment. The area boxed in each panel indicates the best candidate site for a short diagnostic amplicon flanked by conserved primer sites, between nucleotides 160bp and 380bp of the alignment.

Click here to access/download
Supplementary Material
Additional_file_1_12S_Database_sequences.fasta

Additional_file_2_12S_Database_samples

Click here to access/download
Supplementary Material
Additional_file_2_12S_Database_samples.csv

Click here to access/download
Supplementary Material
Additional_file_3_RCode_Window_analysis.txt

Click here to access/download
Supplementary Material
Additional_file_4_FULL_Database.fasta

Click here to access/download

Supplementary Material

Additional_file_5_UNIQUE_Database.fasta

Click here to access/download
Supplementary Material
Additional_file_6_RCode_Tests.txt

Click here to access/download
Supplementary Material
Additional_file_7_GeneticDistanceEvaluation_Results.csv

Additional_file_8_LabEvaluation_Samples

Click here to access/download
Supplementary Material
Additional_file_8_LabEvaluation_Samples.csv

Click here to access/download
Supplementary Material
Additional_file_9_KnownScat_sequences.fasta
