# Supplementary material for: A single mini-barcode test to screen for Australian mammalian predators from environmental samples
Source: Gigascience. 2017 Jul 4;6(8):1–13. doi: 10.1093/gigascience/gix052 (PMC5545080; doi:10.1093/gigascience/gix052)
Supplement: Response-to-Reviewer-Comments_Original-Submission.pdf [file gix052_response-to-reviewer-comments_original-submission.pdf]

Author's Response To Reviewer Comments

Responses to the reviewers
all the changes in the text are highlighted in yellow and the line numbers where the changes occurred are noted in the “responses” part of the table below

reviewer 1: Andrea Galimberti

- ROWS 111-114: Use "region" instead of "sequence" and the sentence is quite redundant and somewhat circular. I suggest to rephrase it.

- yes, I agree. I made the sentence shorter. Lines 118-120

- TABLE 1: It is unclear, which criteria were used to adopt the two threshold values. Maybe the authors can calculate a sort of optimum threshold due to minimum cumulative error rate (see Ferri et al. 2009 DOI: 10.1186/1742-9994-6-1or Galimberti et al. 2012 http://dx.doi.org/10.1371/journal.pone.0040122)

- That is what we did, it is based on the thresholds with the lowest cumulative error. I modified the legend to make it clearer.
Lines 193-196
This can be found in the method section, in “Bioinformatic evaluation of the mini-barcode” (lines 454-456) and it is calculated in ◊the additional file 6 for the R code in the section “MODEL” “# Identify the optimal genetic distance threshold for the raw model for "FULL"-“UNIQUE” ”

- ROWS 260-262: This is an important point. What contingency plan the authors propose to overcome this limit? It is unclear from the text.

- I have added “In practice, any such sequences cannot be used to identify the predator with confidence and therefore must be excluded from analysis.”
Lines 293-295

- I also think that the recent review by Galimberti and colleagues (DOI: http://dx.doi.org/10.4404/hystrix-26.1-11347) concerning DNA barcoding on mammalian taxa should be cited.

- thank you for this paper. I included a citation in the “conservation implications” part of the “Discussion” section. I indeed developed this mini-barcode for a management and monitoring purpose thus for a broader application than the “simple” identification purpose. Line 347

reviewer 2: Stephane Boyer

- It is interesting to see that a more relaxed genetic distance threshold may be more appropriate (line 201). The authors used the default 1% threshold in the functions bestCloseMatch and threshID. They seem to base this decision on the graphical representation of threshID (code

below).

```
>barplot(t(threshfullMat) [4:5,],
>names.arg=paste((threshfullMat[,1]*100), "%"))
```

The visual reading of this barplot gives some indication of how many false positives/negatives the user may have to tolerate. However, this is somewhat a crude measure of the optimal threshold. A better option is to use the localMinima function in SPIDER, which calculates the most appropriate threshold to use for a given dataset based on pairwise distances only. When running this function on the full dataset (see code below), I obtained a threshold of 0.0335 which seems more appropriate for the data. The authors may want to re-visit their analysis based on that threshold (instead of 1%).

```
>#local minima calculation of optimal species delineation threshold
>Thresh <- localMinima(fullDist) #Compute the localMinima function
>#Results: 0.0335 ; 0.195
>plot(Thresh, main="localMinima 12S FULL")
```

If the authors choose to use the localMinima function, the optimal threshold should be calculated using the Unique dataset only. As it is not possible to calculate an accurate threshold with this function using singletons only.

- The reviewer is correct, I chose 1% for the FULL database and 4% for the UNIQUE database based on the code lowest cumulative error.

I have not used localMinima, and I thank the reviewer for making us aware of this option. However, this does not seem to provide a sensible output for the unique database in this instance. As suggested, I have used a threshold of 3.5% (rounding up the localMinima result of 0.335) for the full database. I have incorporated this into the analysis by comparing results using thresholds of 1% and 3.5%. For example, using best close match, The higher threshold results in a greater number of correct identifications, but also a greater number of incorrect identification. In contrast a 1% threshold has a higher number of “no ID” results. I have amended the discussion to note that the most appropriate threshold will depend on the management context, and the relative importance of false positive identifications / unidentified samples.

The results for the unique database using localMinima are more problematic.

Using

```
uniThresh <- localMinima(uniqueDist)
uniThresh$localMinima[1] *100
plot(uniThresh)
```

the threshold identified is 19%, which seems extremely high in this context. While this threshold does produce perfect results (all samples correctly identified with best close match / threshID) using our unique dataset, my concern is that sequences from taxa that are not well represented in our database will be at a much greater risk of misidentification with such a relaxed threshold. Hopefully as more reference sequences become available from a wider range of Australian mammals it will be possible to improve this analysis. However, in the meantime I would argue that in most management contexts it would be better for a sample to be ambiguously identified, or to have “no ID” than to be incorrectly identified. For example, working with the full database, I see the following results using best close match with thresholds of 1% and 3.5%

```
> table(bestCloseMatch(fullDist, Sppfull, thresh = 0.01))
correct incorrect no id
147 3 24
```

```
> table(bestCloseMatch(fullDist, Sppfull, thresh = 0.035))
correct incorrect no id
152 6 16
```

And using threshID with the same thresholds I get:

```
> table(threshID(fullDist, Sppfull, thresh = 0.01))
ambiguous correct incorrect no id
5 142 3 24
> table(threshID(fullDist, Sppfull, thresh = 0.035))
ambiguous correct incorrect no id
12 141 5 16
```

To improve consistency between the two sets of results, I have also amended the text so that analyses with the unique database also use a threshold of 3.5%. This has the same cumulative error as a threshold of 4% (which is what was previously used) and the results are not affected by this change.

text added

Methods: lines 456 + 461-462

Results: lines 183-186, 193-196, 201-202, 216-231

Discussion: lines 319-323

Table 1, Additional file 6, Additional file 7

- I can only commend the authors for providing the annotated R code. The main code works well and is easy to follow. The very last line of code seems incomplete. I think it misses a closing bracket at the very end and another line to query a sequence (as written below)

```
>}
>withinF[[1141]]
```

- The reviewer is correct that the code should end this way. However, in my version of the file this text is not missing.

I have uploaded the file again to make sure that there are no errors.

- I was a little confused with the code for sliding window analysis. I don't understand why the window width was set on 20 bp and why only this particular length was investigated. The authors seem to have used the sliding window analysis to determine the position of potential primers, rather than the position of a suitable mini-barcode region (which was the original purpose of sliding window). If that is the case, then I suppose suitable 'primer windows' must be highly conserved, but what were the other criterion used to select them? It reads as follow on line 343: "…regions up to 200 bp in length, incorporating two primer sites (each of 20 bp in length) that were well-conserved across all taxa but which flanked a region of 100-200 bp that displayed high levels of interspecific variation" What is the threshold for 'well conserved'? What is considered 'high levels of interspecific variation'? Are these based on values obtained from the sliding window analysis?

I would have expected that a range of length, for example from 50 bp up to 200 bp, would have been investigated with the aim of determining the shortest possible mini-barcode region. For example, I ran a sliding window analysis using a width of 150 bp (see code below modified from the authors').

```
>a12SWin <- slidingWindow(a12Sref, width = 150, interval=1)
```

```
>length(a12SWin)
>a12SWin[[1]]
>a12SAna <- slideAnalyses(a12Sref, Sppa12S, width = 150, interval =
>"codons", distMeasures = TRUE, treeMeasures = TRUE)
>str(a12SAna)
>plot(a12SAna)
```

Useful variables provided by the sliding window function includes the 'proportion of zero non-conspecific K2P distances'. When this value is 0, the window has enough identification power to tell all species apart. All 150 bp windows starting on base ~90 to ~240 are good picks in this regard. So I do believe the chosen region is probably a good one. But it is unclear why the window starting on position 160 was deemed the best window by the authors

- I agree that this section was unclear and I have amended the manuscript to include more detail. I thank the reviewer for these comments as these have helped to improve my explanation and interpretation.
I did indeed use a wider range of window sizes. I first used larger window sizes (100-175bp) to identify potential mini barcode regions. I then used the shorter window sizes (20-30 bp) to identify conserved sites suitable for primer development within the region of the candidate mini-barcode. The combination of both of these factors (a highly diagnostic sequence and conserved primer sites) are crucial for effective barcode design and adjusting the sliding windows analysis seemed like a good way to identify primer sites.
Using larger windows, I identified regions that may have been good candidate mini-barcodes, except that it was not clear that suitable primer sites were present. By restricting the window size, I was able to clearly identify highly conserved primers as well as the diagnostic mini-barcode regions.
While the broader region (90-240bp) identified by the reviewer using 150bp windows could certainly serve as a mini-barcode, my choice of starting position was driven by the identification of a suitable forward primer sequence, and also with the final length of the amplicon in mind given the location of a suitable reverse primer. While the region from 90 bp is identified as a potential mini-barcode using a window size of 150, I also found a good region between bases ~160-~380. Considering just the smaller window size (for primer design), the region around base 160 was a suitable primer site.
I have updated the text to clarify this and to better explain the approach and criteria.
Methods: lines 375-387, 425-436
Results: lines 163-170
I have also amended the figures to reflect this (Figure 2) and have updated the supplementary R code (additional file 3).

- Now, it is important to note that the actual values on the x-axis on the plots (e.g. Figure 2) are the positions of the first nucleotide of the window. As such, the box drawn on Figure 2 and presented as the 'best candidate site for a short diagnostic amplicon' is slightly misleading because each dot on that graph represents one window. There is also an issue with the positioning of that box as it is clearly not located between positions 160 and 380 as suggested in the legend of Figure 2.

- Indeed, the boxed area was a bit to the right on figure 2, it is fixed now.

Also, I changed the legend in figure 2 to precise that a dot was the window and the x-axis represents the first base of a window.

- Last small comment about the code: I found that on my version of R, there is an issue with object names that start with a number (e.g. 12Sref). Just placing a letter as the first character in the name solves the issue.

- Additional file 3: This problem is now fixed with all names starting with 12S preceded by “db” (for database)

- Lines 41-55 There is no flow between these sentences. They need to be better linked together. As it stands it is rather laborious to read.

- I changed the text so the sentences flow better
Lines 44-55

- Line 77. it is not clear what you mean by 'barcode tests'

- changed to improve clarity of meaning
Line 77-81

- Lines 113-114 need rephrasing to avoid repetition

- changed
Lines 116-120

- lines 114-120. This paragraph follows few sentences where the authors described their study and their taxa. I think it needs to be more clear that here they are back to general statements. Alternatively, these general statements could be placed before the sentence starting with 'Our goal was…'

- changed. I put the general statements (the two common limiting factors) before summarising the findings in this study (our goal was…)
Lines 109-120

- Line 136. I think it would be useful to include citation [2] here as it is the one describing the sliding window analysis in details.

- changed
Line 137

- Line 144. To create the UNIQUE database, I am guessing that the first step was to remove the singletons and THEN to only keep one sequence per haplotype. It would make sense to write these two steps in the correct order.

- yes, that makes sense. Changed

Lines 145-146

- I was also surprised to see that you had singletons in the FULL dataset, given that line 132-133, it is stated that: "Sequences were obtained from GenBank, with additional targeted sequencing conducted for species under-represented in GenBank."
If there were indeed singletons and those species were eliminated, it would be useful to list which species they were

- The singletons referred to non-target species. I focused the additional sequencing on specific target taxa most relevant to wildlife surveys in Australia, in particular the quolls which are poorly represented in sequencing databases. Line 133: to specify that I added sequences from the target animals
I amended Additional file 7 to note all singleton species

- Line 205. Yes, but a 5% distance threshold would have caused much ambiguity for the identification of the other sequences. Any chances one of the sequences for Dasycercus cristicauda was obtained on Genbank and could be either mis-identifiation or a different (cryptic) species?

- the two Dasycercus sequences are from the same team of scientists. The origin of only one sequence (AF009889) was mentioned (the Tanami desert in Northern Territory). As for the second, they don't know the origin. So it might be an ID error. I put a note in the text
Lines 216-219
Same was true for a western quoll sequence, lines 183-186

- Line 208. rather than 'a wide range of Australian mammals', please provide the number of species

- changed to note that 40 species were included, but also to emphasise that these represent a wide taxonomic range (ie not just 40 species from a single order).
Line 234-239

- Line 201. Add "and possibly beyond" to the end of the sentence or something similar to acknowledge that you also successfully used the primers with non-mammalian vertebrates. Alternatively, remove reptile amphibian and bird from the previous sentence, and write a new sentence at the end of the paragraph, stating why the primer was tested on those non-mammalian specimens.

- Changed to "This demonstrates the broad applicability of the primers across the mammalian taxa and their potential applicability to other vertebrate classes"
Lines 234-239

- Table 2. The title for this table could be improved. It does not give much information about what the numbers are. To understand this, the reader need to go to the legend and then guess what 'CT' means or go all the way to the list of abbreviations. Depending on where this list sits in the paper, I would advise to state what CT means in the legend of Table 2.

- Changed. Table 2: Add information in the title, and in the legend: described what CT was and how it is calculated.
Line 253
Lines 254-259

- Line 236. I would replace 'the known predator' by 'known predators'

- changed
Lines 261-262

- Line 254-257. Here the authors highlight how their study brings new knowledge in the subject of DNA-based species detection. This is crucial but not extremely clear. Maybe these sentences need to be restricted to 'studies aiming at identifying predators from scat samples'.

- changed to "Previous studies, based on species identification from scats or hairs, have applied barcoding methods to detect individual species across multiple time points (examples in (Fernández et al. 2006), (McKelvey et al. 2006)). Here we have shown that it is also possible to identify multiple species from a single DNA test, using a straightforward PCR and Sanger sequencing approach"
Lines 279-282

- Line 239. 92% amplification success is quite good. It would have been interesting to compare this to what can be obtained with primers targeting longer DNA fragments. I understand this was not the aim of this particular paper, but in a sense the authors went into all the trouble of designing mini-barcodes because 'regular (longer) barcodes' don't work. It would be good to put this 92% success rate into perspective with the success rate of longer barcodes if there was any such data in the literature. It is eluded to on line 277, but the actual numbers are not provided.

- in [41]: 79% of sequences were amplified using a 134 bp fragment, and in [56]: <70% using regions from 243 bp to 708 bp (different regions for different taxa)
Lines 304-307

- Line 273. I would replace 'by' with 'in'

- changed
Line 300

- Lines 277-282. I would be careful not to inflate the implications of the paper. The 'approach' used is simply DNA barcoding, the benefits of which have been widely demonstrated elsewhere. The real novelty lies in the primers and the mini-barcode designed for Australian mammals, which does make a very useful tool for managers and scientists. So rather than the 'approach' I would highlight the primers or the mini-barcode here

- changed to "Using our mini-barcode, DNA can be screened for the presence of multiple Australian predator species in a single and inexpensive test, without the need to develop and

apply a set of species-specific primers for each predator of interest. We provide a non-invasive instrument with potential utility for scientists or managers working with endangered or invasive Australian predators, but a similar approach could be used to target predator assemblages in other regions."
so more focussed on Australia and the development of the mini-barcode than on the barcoding itself
lines 307-312

- Line 278. Replace 'screen' by 'screened'

- changed
Line 307

- Line 299. A reference at the end of this sentence would be useful

- yes, I added 2
Line 333

- Line 329-331. Very interesting potential application

- yes indeed

- Line 514. Keith Crandall was editor, not co-author, on that paper. The citation needs to be modified accordingly

- Changed
Line 562

references:
Fernández N, Delibes M, Palomares F (2006) Landscape evaluation in conservation: molecular sampling and habitat modeling for the Iberian lynx. Ecol Appl 16:1037–1049.
McKelvey KS, Kienast JVON, Aubry KB, et al (2006) DNA analysis of hair and scat collected along snow tracks to document the presence of Canada lynx. Wildl Soc Bull 34:451–455.
