# Supplementary material for: A single mini-barcode test to screen for Australian mammalian predators from environmental samples
Source: Gigascience. 2017 Jul 4;6(8):1–13. doi: 10.1093/gigascience/gix052 (PMC5545080; doi:10.1093/gigascience/gix052)
Supplement: Reviewer-2-Report-(Original-Submission).pdf [file gix052_reviewer-2-report-(original-submission).pdf]

Reviewer Report

Title: A single mini-barcode test to screen for Australian mammalian predators from environmental samples

Version: Original Submission **Date:** 03 May 2017

Reviewer name: Stephane Boyer

Reviewer Comments to Author:

This manuscript by Modave et al. presents a cost effective DNA barcoding tool for wildlife management and biodiversity conservation. The authors have designed new primers for detecting and identifying all mammalian species in Australia from scat samples. Primers were then tested on a subset of tissue samples, museum samples and scat samples and seem to perform well even at low DNA concentration. The method used is sound and well described, although there are few points that need clarification particularly with regards to 1)the choice of the species delimitation threshold and 2)the pinpointing of the best possible mini-barcode. I detail these two points below along with some more minor comments. Overall, I think the manuscript is well written and is a valuable contribution. I expect that my comments/concerns will be relatively easy to address and therefore recommend Minor revision.

Main comments

1. It is interesting to see that a more relaxed genetic distance threshold may be more appropriate (line 201). The authors used the default 1% threshold in the functions bestCloseMatch and threshID. They seem to base this decision on the graphical representation of threshID (code below).

```
>barplot(t(threshfullMat) [4:5,], names.arg=paste((threshfullMat[,1]*100), "%"))
```

The visual reading of this barplot gives some indication of how many false positives/negatives the user may have to tolerate. However, this is somewhat a crude measure of the optimal threshold. A better option is to use the localMinima function in SPIDER, which calculates the most appropriate threshold to use for a given dataset based on pairwise distances only. When running this function on the full dataset (see code below), I obtained a threshold of 0.0335 which seems more appropriate for the data. The authors may want to re-visit their analysis based on that threshold (instead of 1%).

```
>#local minima calculation of optimal species delineation threshold
>Thresh <- localMinima(fullDist) #Compute the localMinima function
>#Results: 0.0335 ; 0.195
>plot(Thresh, main="localMinima 12S FULL")
```

If the authors choose to use the localMinima function, the optimal threshold should be calculated using

the Unique dataset only. As it is not possible to calculate an accurate threshold with this function using singletons only.

2. I can only commend the authors for providing the annotated R code. The main code works well and is easy to follow. The very last line of code seems incomplete. I think it misses a closing bracket at the very end and another line to query a sequence (as written below)

```
>}
>withinF[[1141]]
```

I was a little confused with the code for sliding window analysis. I don't understand why the window width was set on 20 bp and why only this particular length was investigated. The authors seem to have used the sliding window analysis to determine the position of potential primers, rather than the position of a suitable mini-barcode region (which was the original purpose of sliding window). If that is the case, then I suppose suitable 'primer windows' must be highly conserved, but what were the other criterion used to select them? It reads as follow on line 343: "…regions up to 200 bp in length, incorporating two primer sites (each of 20 bp in length) that were well-conserved across all taxa but which flanked a region of 100-200 bp that displayed high levels of interspecific variation"
What is the threshold for 'well conserved'? What is considered 'high levels of interspecific variation'? Are these based on values obtained from the sliding window analysis?

I would have expected that a range of length, for example from 50 bp up to 200 bp, would have been investigated with the aim of determining the shortest possible mini-barcode region. For example, I ran a sliding window analysis using a width of 150 bp (see code below modified from the authors').

```
>a12SWin <- slidingWindow(a12Sref, width = 150, interval=1)
>length(a12SWin)
>a12SWin[[1]]
>a12SAna <- slideAnalyses(a12Sref, Sppa12S, width = 150, interval = "codons", distMeasures = TRUE,
treeMeasures = TRUE)
>str(a12SAna)
>plot(a12SAna)
```

Useful variables provided by the sliding window function includes the 'proportion of zero non-conspecific K2P distances'. When this value is 0, the window has enough identification power to tell all species apart. All 150 bp windows starting on base ~90 to ~240 are good picks in this regard. So I do believe the chosen region is probably a good one. But it is unclear why the window starting on position 160 was deemed the best window by the authors.

Now, it is important to note that the actual values on the x-axis on the plots (e.g. Figure 2) are the positions of the first nucleotide of the window. As such, the box drawn on Figure 2 and presented as the

'best candidate site for a short diagnostic amplicon' is slightly misleading because each dot on that graph represents one window. There is also an issue with the positioning of that box as it is clearly not located between positions 160 and 380 as suggested in the legend of Figure 2.

Last small comment about the code: I found that on my version of R, there is an issue with object names that start with a number (e.g. 12Sref). Just placing a letter as the first character in the name solves the issue.

Minor comments

Lines 41-55 There is no flow between these sentences. They need to be better linked together. As it stands it is rather laborious to read.

Line 77. it is not clear what you mean by 'barcode tests'

Lines 113-114 need rephrasing to avoid repetition

lines 114-120. This paragraph follows few sentences where the authors described their study and their taxa. I think it needs to be more clear that here they are back to general statements. Alternatively, these general statements could be placed before the sentence starting with 'Our goal was…'

Line 136. I think it would be useful to include citation [2] here as it is the one describing the sliding window analysis in details.

Line 144. To create the UNIQUE database, I am guessing that the first step was to remove the singletons and THEN to only keep one sequence per haplotype. It would make sense to write these two steps in the correct order.
I was also surprised to see that you had singletons in the FULL dataset, given that line 132-133, it is stated that: "Sequences were obtained from GenBank, with additional targeted sequencing conducted for species under-represented in GenBank."
If there were indeed singletons and those species were eliminated, it would be useful to list which species they were.

Line 205. Yes, but a 5% distance threshold would have caused much ambiguity for the identification of the other sequences. Any chances one of the sequences for Dasycercus cristicauda was obtained on Genbank and could be either mis-identifiation or a different (cryptic) species?

Line 208. rather than 'a wide range of Australian mammals', please provide the number of species.

Line 201. Add "and possibly beyond" to the end of the sentence or something similar to acknowledge

that you also successfully used the primers with non-mammalian vertebrates. Alternatively, remove reptile amphibian and bird from the previous sentence, and write a new sentence at the end of the paragraph, stating why the primer was tested on those non-mammalian specimens.

Table 2. The title for this table could be improved. It does not give much information about what the numbers are. To understand this, the reader need to go to the legend and then guess what 'CT' means or go all the way to the list of abbreviations. Depending on where this list sits in the paper, I would advise to state what CT means in the legend of Table 2.

Line 236. I would replace 'the known predator' by 'known predators'.

Line 254-257. Here the authors highlight how their study brings new knowledge in the subject of DNA-based species detection. This is crucial but not extremely clear. Maybe these sentences need to be restricted to 'studies aiming at identifying predators from scat samples'.

Line 239. 92% amplification success is quite good. It would have been interesting to compare this to what can be obtained with primers targeting longer DNA fragments. I understand this was not the aim of this particular paper, but in a sense the authors went into all the trouble of designing mini-barcodes because 'regular (longer) barcodes' don't work. It would be good to put this 92% success rate into perspective with the success rate of longer barcodes if there was any such data in the literature. It is eluded to on line 277, but the actual numbers are not provided.

Line 273. I would replace 'by' with 'in'

Lines 277-282. I would be careful not to inflate the implications of the paper. The 'approach' used is simply DNA barcoding, the benefits of which have been widely demonstrated elsewhere. The real novelty lies in the primers and the mini-barcode designed for Australian mammals, which does make a very useful tool for managers and scientists. So rather than the 'approach' I would highlight the primers or the mini-barcode here.

Line 278. Replace 'screen' by 'screened'

Line 299. A reference at the end of this sentence would be useful.

Line 329-331. Very interesting potential application.

Line 514. Keith Crandall was editor, not co-author, on that paper. The citation needs to be modified accordingly.

Methods

Are the methods appropriate to the aims of the study, are they well described, and are necessary controls included? Yes

Conclusions

Are the conclusions adequately supported by the data shown? Yes

Reporting Standards

Does the manuscript adhere to the journal's guidelines on minimum standards of reporting? Yes

Statistics

Are you able to assess all statistics in the manuscript, including the appropriateness of statistical tests used? Yes, and I have assessed the statistics in my report.

Quality of Written English

Please indicate the quality of language in the manuscript: Acceptable

Declaration of Competing Interests

Please complete a declaration of competing interests, considering the following questions:

- Have you in the past five years received reimbursements, fees, funding, or salary from an organisation that may in any way gain or lose financially from the publication of this manuscript, either now or in the future?
- Do you hold any stocks or shares in an organisation that may in any way gain or lose financially from the publication of this manuscript, either now or in the future?
- Do you hold or are you currently applying for any patents relating to the content of the manuscript?
- Have you received reimbursements, fees, funding, or salary from an organization that holds or has applied for patents relating to the content of the manuscript?
- Do you have any other financial competing interests?
- Do you have any non-financial competing interests in relation to this paper?

If you can answer no to all of the above, write 'I declare that I have no competing interests' below. If your reply is yes to any, please give details below.

I declare that I have no competing interests

I agree to the open peer review policy of the journal. I understand that my name will be included on my report to the authors and, if the manuscript is accepted for publication, my named report including any

attachments I upload will be posted on the website along with the authors' responses. I agree for my report to be made available under an Open Access Creative Commons CC-BY license (http://creativecommons.org/licenses/by/4.0/). I understand that any comments which I do not wish to be included in my named report can be included as confidential comments to the editors, which will not be published.

I agree to the open peer review policy of the journal

To further support our reviewers, we have joined with Publons, where you can gain additional credit to further highlight your hard work (see: https://publons.com/journal/530/gigascience). On publication of this paper, your review will be automatically added to Publons, you can then choose whether or not to claim your Publons credit. I understand this statement.

Yes
